# Do’s and Don’ts in Mealtime Management for People with Oropharyngeal Dysphagia: A Modified Delphi Consensus Study from the Italian Society of Deglutology

**DOI:** 10.3390/nu18193268

**Published:** 2026-10-04

**Authors:** Nicole Pizzorni, Sara Rocca, Aurora Ninfa, Antonio Amitrano, Arianna Valeria Bai, Michela Barichella, Maria Rosaria Barillari, Umberto Barillari, Jessica Barsotti, Irene Battel, Monica Bianchi, Alessandra Bosetti, Paola Castellini, Federica Cenci, Emanuele Cereda, Anna Colombo, Antonella Cusimano, Francesco D’Abrosca, Ramona De Amicis, Claudia Dellavia, Antonella Delle Fave, Stefano Doratiotto, Daniele Farneti, Bruno Fattori, Maria Luisa Fiorella, Alessandro Galazzi, Federica Lucia Galli, Elisabetta Genovese, Luisa Gervasio, Silvia Gnecchi, Isabella Koch, Valentina Lamon, Patrizia Maruzzi, Benito Michelizza, Maria Giulia Montecchi, Luca Morelli, Francesco Mozzanica, Luca Parisi, Carlo Pedrolli, Gaia Pellegrini, Chiara Piscitelli, Marco Ramaioli, Cristina Reverberi, Sergio Riso, Giovanni Ruoppolo, Amelia Santoro, Alessandro Selvagio, Massimo Spadola Bisetti, Chiara Stracquadanio, Beatrice Travalca Cupillo, Stefano Usai, Elvira Verduci, Irene Vernero, Lucia Zannini, Antonio Schindler

**Affiliations:** 1 Department of Biomedical and Clinical Sciences, University of Milan, 20157 Milan, Italy; nicole.pizzorni@unimi.it (N.P.); antonio.schindler@unimi.it (A.S.); 2 Phoniatric and Speech and Language Pathologist Unit, Vittore Buzzi Children’s Hospital, 20154 Milan, Italy; aurora.ninfa@unimi.it (A.N.); anna.colombo89@asst-fbf-sacco.it (A.C.); patmaruzzi@libero.it (P.M.); selvagio.alessandro@asst-fbf-sacco.it (A.S.); 3 Degree Courses in Health Professions “Luigi Sacco” Hospital, University of Milan, 20157 Milan, Italy; 4 Independent Researcher, 20154 Milan, Italy; a.amitrano@yahoo.it; 5 Developmental Neuroscience Clinical Department, IRCCS Fondazione Stella Maris, 56128 Pisa, Italy; ariannavaleria.bai@fsm.unipi.it; 6 Clinical Nutrition Unit, ASST Gaetano Pini-CTO, 20126 Milan, Italy; michela.barichella@asst-pini-cto.it; 7 Fondazione Pezzoli per la Malattia di Parkinson, 20125 Milan, Italy; 8 Division of Phoniatrics and Audiology, Department of Mental and Physical Health and Preventive Medicine, University of Campania “Luigi Vanvitelli”, 80138 Naples, Italy; mariarosaria.barillari@unicampania.it (M.R.B.); umbertoe.s.barillari@unicampania.it (U.B.); 9 Department of Child Psychiatry and Psychopharmacology, IRCCS Stella Maris Foundation, 56128 Pisa, Italy; jessica.barsotti@fsm.unipi.it; 10 Department of Biomedical, Surgical and Dental Sciences, University of Milan, 20122 Milan, Italy; irene.battel@gmail.com (I.B.); claudia.dellavia@unimi.it (C.D.); luca.parisi@unimi.it (L.P.); gaia.pellegrini@unimi.it (G.P.); 11 Department of Business Economics, Health and Social Care, University of Applied Sciences and Arts of Southern Switzerland (SUPSI), CH-6928 Manno, Canton Ticino, Switzerland; monica.bianchi@supsi.ch; 12 Department of Pediatrics, Vittore Buzzi Children’s Hospital, 20154 Milan, Italy; alessandra.bosetti@asst-fbf-sacco.it; 13 Phoniatric Unit, IRCCS AOM-Policlinico San Martino, 16132 Genoa, Italy; paolacastellini65@gmail.com (P.C.); antonella.cusimano.dott@gmail.com (A.C.); 14 Occupational Therapy Service, Intermediate Care Unit, Health and Social Care Department, ASP Golgi-Redaelli, 20146 Milan, Italy; federica.cenci@golgiredaelli.it; 15 Clinical Nutrition and Dietetics Unit, Fondazione IRCCS San Matteo, 27100 Pavia, Italy; e.cereda@smatteo.pv.it; 16 Department of Health Sciences, University of Eastern Piedmont Amedeo Avogadro, 28100 Novara, Italy; fradabro@gmail.com; 17 Department of Food, Environmental and Nutritional Sciences (DeFENS), University of Milan, 20133 Milan, Italy; ramona.deamicis@unimi.it; 18 Department of Endocrine and Metabolic Diseases, Obesity Unit and Laboratory of Nutrition and Obesity Research, IRCCS Istituto Auxologico Italiano, 20145 Milan, Italy; 19 Department of Pathophysiology and Transplantation, University of Milan, 20122 Milan, Italy; antonella.dellefave@unimi.it; 20 Department of Diagnostic and Interventional Radiology, Ca’ Foncello Hospital, 31100 Treviso, Italy; stefano.doratiotto@aulss2.veneto.it; 21 Audiologic Phoniatric Service, ENT Department AUSL Romagna, Infermi Hospital, 47923 Rimini, Italy; daniele.farneti@auslromagna.it; 22 ENT, Audiology, and Phoniatrics Unit, University Hospital of Pisa, University of Pisa, 56124 Pisa, Italy; bruno.fattoriorl@gmail.com (B.F.); asantoro_dr@hotmail.com (A.S.); 23 Otolaryngology Unit, Department of Biomedical Sciences, Neuroscience and Sensory Organs, University of Bari “Aldo Moro”, 70124 Bari, Italy; marialuisa.fiorella@uniba.it; 24 Department of Medicine and Surgery, LUM University, 70010 Casamassima, Italy; galazzi@lum.it; 25 Department of Experimental and Clinical Medicine, Marche Polytechnic University, 60126 Ancona, Italy; f.l.galli@staff.univpm.it; 26 Former, Audiology Program, Department of Maternal, Child and Adult Medical and Surgical Sciences, University of Modena and Reggio Emilia, 41124 Modena, Italy; elisabetta.genovese@unimore.it; 27 Pharmacy Service, I.R.C.C.S. Foundation Istituto Neurologico Nazionale C. Mondino, 27100 Pavia, Italy; luisa.gervasio@mondino.it; 28 IRCCS San Raffaele Scientific Institute, 20132 Milan, Italy; gnecchi.silvia@hsr.it; 29 Independent Researcher, Lido, 30126 Venice, Italy; isabellalogo@gmail.com; 30 Clinical Nutrition Unit, ASST Fatebenefratelli Sacco, 20157 Milan, Italy; valentina.lamon@asst-fbf-sacco.it; 31 Istituto Molisano di Riabilitazione, 86039 Termoli, Italy; benito.michelizza@gmail.com; 32 Neurorehabilitation Unit, Dipartimento Neuromotorio Riabilitativo, Ospedale San Sebastiano, Correggio, Ausl-IRCCS Reggio Emilia, 42123 Reggio Emilia, Italy; mariagiulia.montecchi@ausl.re.it; 33 Department of Audiovestibology—Cochlear Implant Center, ASST Sette Laghi, 21100 Varese, Italy; luca.morelli@asst-settelaghi.it; 34 Department of Otorhinolaryngology, IRCCS Multimedica, Ospedale San Giuseppe, 20123 Milan, Italy; francesco.mozzanica@unimi.it; 35 Department of Clinical Sciences and Community Health (Department of Excellence 2023–2027), University of Milan, 20122 Milan, Italy; 36 Azienda Sanitaria Universitaria Integrata del Trentino (ASUIT), Santa Chiara Hospital, 38122 Trento, Italy; carlo.pedrolli@asuit.tn.it; 37 Neonatal and Pediatric Department, ASL Roma 6, 00041 Albano Laziale, Italy; logopedistachiarapiscitelli@gmail.com; 38 Université Paris-Saclay, INRAE, AgroParisTech, UMR SayFood, 91120 Palaiseau, France; marco.ramaioli@inrae.fr; 39 Azienda USL IRCSS di Reggio Emilia, 42123 Reggio Emilia, Italy; cristina.reverberi@unimore.it; 40 Dietetic and Clinical Nutrition Unit, Azienda Ospedaliero-Universitaria Maggiore della Carità, 28100 Novara, Italy; sergio.riso@alice.it; 41 Former, Sapienza Università di Roma, 00185 Rome, Italy; giovannialfonso.ruoppolo@gmail.com; 42 A.O.U. Città della Salute e della Scienza di Torino, 10126 Turin, Italy; spadola.massimo@alice.it; 43 ASST GOM Niguarda, 20162 Milan, Italy; chiara.stracquadanio@ospedaleniguarda.it; 44 Former, Phoniatric Unit, IRCCS AOM-Policlinico San Martino, 16132 Genoa, Italy; beatrice.travalca@gmail.com; 45 Centro Studi Riabilitazione Neurocognitiva di Villa Miari, 36014 Santorso, Italy; stefano.usai@hotmail.com; 46 Department of Health Sciences, University of Milan, 20146 Milan, Italy; elvira.verduci@unimi.it; 47 Pediatric Unit, Foundation IRCCS Ca’ Granda Ospedale Maggiore Policlinico, 20122 Milan, Italy; 48 Former, University of Turin, 10124 Turin, Italy; irene.vernero@gmail.com; 49 Department of Biomedical Sciences for Health, University of Milan, 20133 Milan, Italy; lucia.zannini@unimi.it

**Keywords:** deglutition disorders, deglutition, meal, nutritional sciences, Delphi technique

## Abstract

**Background:** Meals are central to daily life, health, and social participation, yet their safety and enjoyment can be compromised in people with oropharyngeal dysphagia (OD). Despite the broad impact of OD on physical and psychosocial well-being and daily functioning, structured guidance for comprehensive mealtime management in clinical practice is lacking. To address this gap, a set of expert consensus-based “Do’s and Don’ts” covering four meal-related domains was developed. **Methods:** A modified Delphi consensus study promoted by the Italian Society of Deglutology involved a multidisciplinary panel of 53 experts in OD and related disciplines. Experts developed “Do” and “Don’t” statements across four domains: nutrition-focused diet prescription, meal preparation, mealtime experience, and patient and caregiver education. After panel conveners’ review, 61 statements underwent two rounds of online voting using a 3-point ordinal scale plus an additional option ‘unable to express an opinion’. Consensus was predefined as ≥75% agreement. Statements were revised between rounds based on participants’ comments to ensure clinical accuracy and practical applicability. **Results:** The response rate was 85% for Round 1 and 97.8% for Round 2. After Round 2, 57 of 61 statements (93.4%) reached the consensus threshold (≥75% agreement). **Conclusions:** Mealtime management in OD should be individualized and person-centered, balancing swallowing safety, nutritional requirements, quality of life, and enjoyment of eating. Management should extend beyond compensatory strategies alone to include the social and relational dimensions of meals. Active involvement and education of patients and caregivers are essential to support therapeutic adherence and daily implementation of recommendations. Interprofessional collaboration and multimodal educational strategies are crucial to ensuring a coordinated, safe, and effective nutritional approach to OD care.

## 1. Introduction

Eating and drinking are fundamental activities in our lives. Meals rule the organization of our days, reflect a person’s social, cultural, religious, and geographical background, and, when shared with others, symbolize family, friendship, and community. However, the safety and enjoyment of meals may be disrupted in people living with oropharyngeal dysphagia (OD). OD is defined as a “swallowing dysfunction that causes impairment in moving the bolus safely and effectively from the mouth through the pharynx into the esophagus” [1]. OD is a highly prevalent condition across the lifespan. A recent meta-analysis reported a pooled OD prevalence of 13.4% (95% confidence interval (CI) 4.4–34.5) in the general population, with higher rates among older adults (48.1%, 95% CI 31.9–64.7), and markedly higher prevalence in individuals with conditions such as dementia (72.4%, 95% CI 26.7–95.0) and stroke (55.4%, 95% CI 37.2–72.2) [2]. In the pediatric population, OD can be caused by congenital conditions, developmental disorders, extreme prematurity and other complex medical conditions and syndromes. In the adult population, a wide range of diseases can lead to OD, including neurological, neuromuscular, rheumatological, endocrinological, infectious, structural, and oncological conditions, as well as clinical procedures such as intubation [1]. Additionally, aging-related neurological and anatomical changes can expose older people to an increased risk of OD [3].

Meals of people with OD are often characterized by prolonged duration, fatigue, reduced intake of nutrients, difficulties in oral preparation and mastication, feeling of food stuck in the throat, cough, and even choking. These problems entail risks of pulmonary complications, malnutrition and dehydration [4,5,6,7,8,9]. As malnutrition is a critical determinant of clinical outcomes, the dietetic approach must ensure that nutritional requirements are met, even in the presence of restrictive swallowing modifications. A meta-analysis including 39 studies and 31,488 older adult participants showed that the presence of OD doubled the risk of pneumonia and malnutrition [2]. Swallowing safety at meals was found to be an independent predictor of malnutrition in patients with neurodegenerative diseases [10]. Similar challenges exist in the pediatric population, where OD is frequently associated with lower daily energy and fluid intake, directly impacting growth [11,12,13].

Along with health complications, people with OD can experience negative affect, poor quality of life, and reduced social participation [14,15,16,17,18]. Psychosocial consequences can also impact the lives of caregivers and family members [19,20]. A systematic review revealed an increased burden in 70% of caregivers of people with OD, mainly related to changes in meal preparation, disruption in lifestyle, effects on social life, and lack of support [21]. Care needs of people living with OD and their caregivers span physical, practical, informational, social, and psychological aspects [22]. When OD arises, needs include the acquisition of knowledge and skills to manage it in daily living. For instance, caregivers should learn how to manage the complex logistics of mealtimes, including the need to provide meals that are both safe and nutritionally adequate. In two qualitative studies involving people with chronic OD and their family members, daily challenges related to meal management were reported by one in two informal caregivers and one in three patients [23,24]. In caregivers of post-stroke survivors, caregiving burden was significantly associated with the perceived impact of OD on mealtime logistics [25]. This evidence calls on healthcare services to address these needs to promote patients’ and families’ self-management, quality of life, and social participation.

In clinical practice and research, OD assessment and management are primarily focused on swallowing as a body function [26,27]. A scoping review mapping the research on OD rehabilitation among head and neck cancer patients highlighted that study outcomes predominantly fell within the definition of impairment from the International Classification of Functioning, Disability and Health (ICF) [27]. The ICF, developed by the World Health Organization, is a comprehensive framework for describing and organizing information on functioning and health [28]. It relies on a biopsychosocial model which integrates medical and social perspectives, to conceptualize health as the result of interactions between an individual’s health condition and contextual factors. The ICF classifies functioning across three domains: body functions and structures, activities, and participation, while also considering environmental and personal factors that can act as barriers or facilitators. Several studies have mapped ICF codes related to OD [29,30,31], highlighting the importance of broadening healthcare’s view on OD from body structures and functions to other ICF domains. Wu and colleagues ranked various ICF codes associated with OD according to their impact on individual functioning [32]. Notably, the categories related to social participation—“Ceremonies,” “Hobbies,” and “Socializing”—were assigned the highest positions, indicating that these domains may constitute particularly meaningful targets for clinical intervention.

Shifting from swallowing management to meal management may be of particular importance in some clinical conditions. Firstly, swallowing function cannot be significantly improved in several conditions such as the advanced stages of neurodegenerative diseases, late-onset dysphagia after (chemo)-radiotherapy for head and neck cancer [33], or Spinal Muscular Atrophy in children [34]. Yet, improving meal management may be an intervention target even in these populations. Secondly, levels of swallowing capacity observed in the clinical setting may not always overlap with swallowing performance during daily meals. For instance, cognitive and behavioral symptoms may negatively affect swallowing safety and efficiency at meals. In these cases, the adoption of adequate strategies, the allocation of attentional resources, and the control over environmental disrupting factors during meals may facilitate swallowing performance. Thus, a comprehensive overview of each individual’s functioning in everyday life is essential to provide patients and families with feasible and implementable recommendations. This is particularly relevant in light of the frequently reported low levels of adherence to medical and behavioral prescriptions to manage OD [35,36].

Formal, evidence-based clinical practice guidelines, typically developed through systematic literature review and structured evidence grading, represent a well-established approach to informing clinical recommendations [37]. However, this approach presupposes a sufficiently developed evidence base, a condition seldom met for many practical, day-to-day aspects of mealtime management in OD for which expert opinion is often the only source of evidence. A modified Delphi approach, a structured consensus method, was therefore considered the most suitable method to systematically collect, refine, and aggregate the collective clinical judgement of a large, multidisciplinary panel of professionals involved in the care of people with OD [38,39].

Nevertheless, to the best of our knowledge no structured guidelines are currently available to support health professionals in helping people with OD to manage mealtimes, ensuring safety and efficiency while preserving enjoyment, an area characterized by considerable heterogeneity in available evidence and clinical practice. The aim of this study was therefore to develop, through a modified Delphi process involving experts from the Italian Society of Deglutology, a set of expert consensus statements on do’s and don’ts for mealtime management in people with OD. Specifically, four domains were identified: nutrition-focused diet prescription, meal preparation, mealtime experience in daily life, and patient and caregiver education. Importantly, this study was designed to capture professional judgement and expert agreement on suggested practice, and not to document professionals’ actual behaviors in clinical practice, nor to test the clinical effectiveness of the resulting statements, which will require dedicated future research.

## 2. Materials and Methods

**Figure 1:**
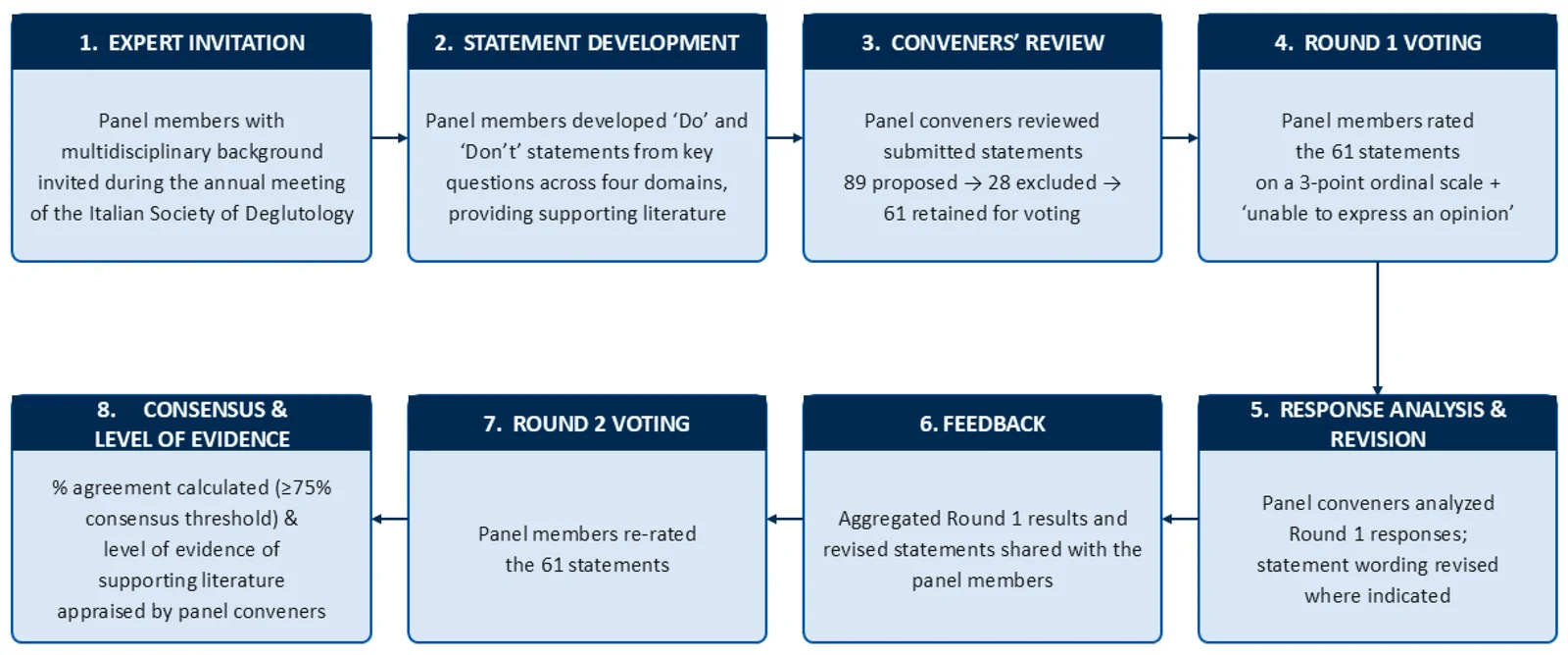
Flowchart of the stages of the modified Delphi process.

A modified Delphi method was used. The Delphi technique is a well-established, systematic approach for eliciting and refining expert opinion and achieving consensus, particularly in areas where empirical evidence is limited or heterogeneous [40]. The study was approved by the Ethics Committee of the University of Milan (approval number: 21/25). The study protocol was not registered. All panel members provided written informed consent to participate. This study is reported in accordance with the CREDES (Conducting and REporting of DElphi Studies) checklist [41]. Study procedures are illustrated in Figure 1.

### 2.1. Panel

The modified Delphi consensus process was initiated during the annual meeting of the Italian Society of Deglutology, held in Milan on 25–26 October 2024. The course, entitled *“Mealtime Management in People with Dysphagia Across the Lifespan: From Birth to 100 Years,”* brought together 53 nationally recognized experts in deglutology and related disciplines. Participants represented a broad range of professionals, including speech and language pathologists, phoniatricians, otorhinolaryngologists (ENT specialists), physicians specialized in clinical nutrition, dietitians, physiatrists, physiotherapists, occupational therapists, dentists, dental hygienists, nurses, radiologists, pharmacists, food technologists, psychologists, and experts in educational sciences. Characteristics of the panel are reported in Table 1.

Experts were selected based on at least one of the following criteria: (a) being an academic researcher or faculty member at a national or international university or research institution; (b) being a current or former board member of a national or international scientific society in deglutology or related disciplines; or (c) having authored scientific publications in deglutology or related fields.

All experts were invited to participate in the Delphi study as panel members. Four of them served as panel conveners and were responsible for the design and conduct of the Delphi process. Regarding sample size, an optimal panel should typically comprise between 10 [42] and 50 [43,44] experts, depending on the complexity of the topic and the desired heterogeneity of the panel [45]. Given that attrition between Delphi rounds is common [38] and considering the need for a multidisciplinary and multiprofessional panel to address meal management in OD, a sample of 53 experts was deemed appropriate and consistent with published recommendations.

### 2.2. Question Development

The panel conveners identified four key domains relevant to the clinical and everyday management of mealtimes in people with OD: diet prescription, meal preparation, mealtime experience in daily life, and patient and caregiver education. Within each domain, one or two clinically relevant questions were developed to guide statement generation, focusing on areas characterized by variability in practice and limited or heterogeneous evidence (Table 2).

### 2.3. Statement Development

Each expert was assigned to one clinical question and invited to develop one or two ‘Do’ and one or two ‘Don’t’ statements addressing the question, supported by relevant references, following the methodology used in a recent consensus on head and neck cancer [46]. Proposing experts were asked to consult PubMed, Embase, and Scopus to identify literature supporting their proposed statements, with the option to include additional relevant references identified outside these databases. No standardized search strategy, explicit selection criteria, or formal appraisal of the quality or risk of bias of the identified references were applied; literature identification and selection were performed individually by each expert according to their own clinical and academic judgment. ‘Do’ statements denote recommended clinical actions informed by available evidence and clinical experience. ‘Don’t’ statements denote actions that should be avoided, as they may be ineffective, inappropriate, or potentially harmful. Crucially, the statements were developed to reflect a multidisciplinary perspective, incorporating the specific roles of nutritionists/dietitians, speech-language pathologists, nurses, physiotherapists, occupational therapists, and medical specialists in meal management. Experts were asked to avoid generic statements on OD management unless they specifically pertained to meal management. Statement development was informed by available scientific evidence and expert clinical judgment. In areas where evidence was lacking or heterogeneous, statements were primarily based on expert opinion and current best practices.

All proposed statements were submitted to the panel conveners, who reviewed them for relevance, specificity, and actionability. Statements considered too broad or not directly applicable to mealtime management were excluded. The conveners also harmonized the wording of the retained statements to ensure consistency and clarity, without altering their clinical meaning. A total of 89 statements were initially submitted, of which 28 were excluded after review. Statements excluded from voting, along with the corresponding reasons for exclusion, are reported in the Supplementary Material S1. Reasons for exclusion included lack of specificity to mealtime management or to OD, integration of the statement’s content into another retained statement, redundancy with another statement, and falling outside the aim of the study. Therefore, 61 statements were included in the final Delphi process for consensus evaluation. Based on the references provided by the proposing expert for each statement (reported in Supplementary Materials S2), the four panel conveners jointly assigned a level of evidence to the literature supporting each statement according to the Infectious Diseases Society of America–US Public Health Service grading system that was adapted by the European Society for Medical Oncology [47]. The grading system ranged from I (high level of evidence) to V (low level of evidence). When supporting literature served as rationale for statement development without directly supporting its specific content or was not specific to people with OD, a level of evidence V was assigned.

### 2.4. Delphi Consensus Process

Prior to the initiation of the Delphi consensus process, the panel conveners predefined that the statements would be subjected to two rounds of voting. Delphi studies typically comprise two or three rounds [48], as additional rounds are associated with higher attrition rates and may increase the risk of forced consensus [49].

Panel membership was not anonymous, as participants were recruited during the annual meeting of the Italian Society of Deglutology. However, the identity of the expert who had proposed each statement was not disclosed to other panelists, and individual voting responses remained anonymous to other participants throughout both rounds. Only the panel conveners had access to both statement authorship and individual voting records, the latter being necessary to track participation across rounds. Experts voted on all statements, including those they had proposed themselves. Panel conveners were also part of the voting panel.

Experts rated each statement using a 3-point ordinal agreement scale (‘agree’, ‘agree with reservations’, ‘disagree’), with an additional option ‘unable to express an opinion’. When selecting ‘agree with reservations’ and ‘disagree’, participants were asked to provide a rationale in an open-ended response and were allowed to edit the statements. Consensus was defined a priori as the proportion of ‘agree’ and ‘agree with reservations’ responses relative to all respondents, including those who selected “unable to express an opinion.” This approach was adopted as a conservative choice, avoiding an inflated consensus percentage that could result from excluding non-substantive responses from the denominator. As a sensitivity analysis, the percentage of agreement for each statement was additionally recalculated excluding “unable to express an opinion” responses from the denominator. The two response categories ‘agree’ and ‘agree with reservations’ were combined because both reflect substantive endorsement of a statement’s clinical content, as distinct from “disagree,” which reflects rejection of the statement itself, consistent with other established consensus methods [50]. Given the absence of a universally accepted threshold for consensus in Delphi studies, with reported values ranging from 50% to 97% and a median of 75% [39], a threshold of ≥75% agreement was considered indicative of consensus for this study.

The survey was administered using Microsoft Forms (Microsoft 365, Microsoft Corporation, Redmond, WA, USA); no pilot testing was performed prior to Round 1. Round 1 was conducted in March 2025 and Round 2 in September 2025. Only experts who completed Round 1 were invited to participate in Round 2. Each round lasted five weeks. All items were retained for Round 2, regardless of the level of agreement achieved in Round 1. Following Round 1, open-ended responses (i.e., rationales for ‘disagree’ and ‘agree with reservations’ ratings, as well as suggested edits) were reviewed by the panel conveners to refine statements ensuring technical accuracy, without altering their original intent. At the beginning of Round 2, participants received the survey link together with a summary report of Round 1 results, including aggregated response distributions and both the original and revised versions of each statement.

## 3. Results

**Figure 2:**
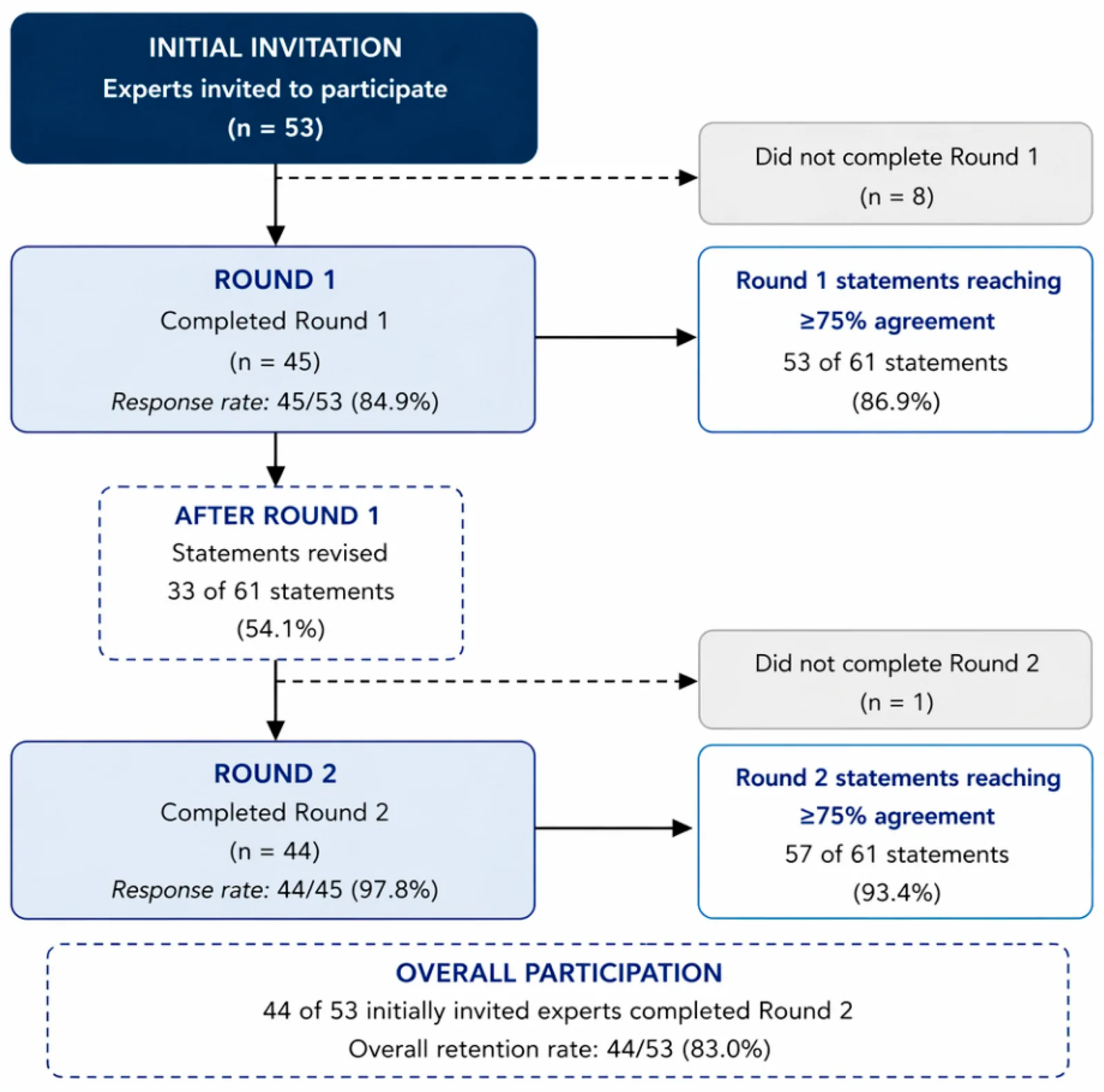
Flow-chart of the Delphi process: response rate and statements reaching the consensus threshold. Note. Response rates are calculated using the number of invited participants for each round as the denominator.

The flowchart of the modified Delphi process, including response rates and the number of statements reaching the predefined consensus threshold (≥75% agreement), is presented in Figure 2. The full list of statements, with agreement levels for each round and the level of evidence of the supporting literature, is reported in Table 3. Forty-seven (77%) statements were classified as Level V (low level of evidence). Frequencies of each response option at each round, and a sensitivity analysis recalculating the percentage of agreement for each statement after excluding “unable to express an opinion” responses from the denominator, are reported in Supplementary Materials S3.

### 3.1. Round 1

The response rate for Round 1 was 85% (45/53 experts). Based on experts’ ratings, 53 out of 61 statements (86.9%) reached the consensus threshold (≥75% agreement). The median level of agreement across statements was 100% (range: 57.8–100%). Following Round 1, 33 statements (54.1%) were revised based on experts’ comments; original and revised statements are reported in Supplementary Material S4.

### 3.2. Round 2

The response rate for Round 2 was 97.8% (44/45 experts invited; overall retention from the initial panel: 83.0%). Based on the experts’ ratings, 57 of the 61 statements (93.4%) reached the consensus threshold (≥75% agreement). Statements #9, #10, #22, and #28 failed to reach the consensus threshold (reported in *italics* in Table 3). The median level of agreement across statements was 100% (range: 63.6–100%). The ‘Do’ and ‘Don’t’ statements meeting the consensus threshold are presented and discussed below.

## 4. Discussion

The present study sought to provide structured, pragmatic and clinically oriented indications for the mealtime management of people with OD. Statements covered multifaceted aspects, from swallowing safety and efficiency to quality of life and the hedonic and social dimensions of eating. Through a modified Delphi process involving experts from the Italian Society of Deglutology, consensus was reached on a series of practical “do’s and don’ts” across four key domains: diet prescription, meal preparation, mealtime experience in daily life, and patient and caregiver education. The statements that achieved expert consensus are discussed below in light of current literature and clinical perspectives. Importantly, these findings extend beyond traditional approaches to dysphagia management by emphasizing the role of mealtimes as complex, context-dependent activities, consistent with biopsychosocial models such as the ICF.

### 4.1. Do’s and Don’ts on Diet Prescription

#### 4.1.1. Swallowing Assessment

##### 4.1.1.1. Clinical and Instrumental Assessment


**DO**

To provide individualized recommendations, bolus trials should follow an ecological criterion that replicates the home mealtime environment as closely as possible, including foods (flavors, colors, smells), consistencies and textures coded using IDDSI terminology, utensils, and environmental supports (e.g., anatomical seating, parent/caregiver presence).When reporting gold standard instrumental swallowing assessments (FEES, VFSS) and clinical evaluations, it is suggested to specify the volumes, consistencies and textures (using IDDSI terminology) of foods and beverages that allow the person with OD to eat with optimal safety and efficiency.During history taking, it is suggested to investigate the modality used to administer medications, including any need to manipulate solid dosage forms due to OD.

**DON’T**

It is suggested not to perform an instrumental evaluation aimed at diet prescription without a prior clinical assessment that clarifies the indications for the exam and suggests the most appropriate safety conditions.


Texture and consistency modifications are widely used in OD management. However, their use should be individualized according to instrumental and/or clinical swallowing assessment and should not be routinely prescribed without a comprehensive evaluation.

Clinical and instrumental evaluations of swallowing represent two distinct yet complementary components of OD assessment across the lifespan. Clinical assessment provides an initial understanding of swallowing safety and efficiency, identifies signs and symptoms suggestive of OD, and informs the need for further investigation [51]. Instrumental assessment allows direct visualization of swallowing physiology, enabling the identification of pathophysiological mechanisms and supporting more precise clinical decision-making [52]. The use of standardized and shared assessment protocols is widely advocated [51,52,53]. Standardization facilitates comparison of data across studies, clinical populations, and settings and enables monitoring of swallowing performance over time. Furthermore, standardized protocols allow the systematic investigation of swallowing physiology across different volumes and consistencies. Finally, the use of standardized protocols during videofluoroscopic swallowing studies (VFSS) may reduce examination time and, consequently, minimize radiation exposure [54].

Despite offering important advantages, standardized approaches may present limitations in guiding individualized dietary and procedural recommendations for everyday life. Standard protocols typically rely on predefined bolus types and controlled conditions, which may not fully reflect the complexity and variability of real-world eating settings. For this reason, it is advisable to complement standardized protocols with more ecologically valid testing conditions. This approach is particularly relevant during clinical swallowing examination and fiberoptic endoscopic evaluation of swallowing (FEES), where there are no radiation-related time constraints and a wider range of foods can be tested. Depending on the clinical questions, the foods and beverage that are easiest or most difficult to swallow, and that the person with OD typically consumes, may be included. Such an approach is especially relevant in pediatric populations, where only a limited number of items can often be tested, making it essential to prioritize foods that are representative of the child’s daily intake [55]. It is also particularly important when evaluating solid foods, which show widely varying physical properties (e.g., hardness, cohesiveness, slipperiness), only partially represented in standard assessment protocols. Similarly, assessment conditions such as utensil use (e.g., cup, straw, spoon, syringe) and posture (e.g., seated in a highchair, fed in a caregiver’s arms) should reflect the individual’s usual mealtime context. Patients with Parkinson’s disease warrant particular consideration, as testing should ideally be performed in ON-drug conditions [56,57]. This ecological approach enhances the clinical relevance of the assessment and supports the formulation of practical, applicable, and individualized recommendations.

Therefore, the selection of the instrumental assessment protocol, beyond any standardized protocol, should be guided by both a thorough history taking (including a detailed review of current dietary intake) and findings from the clinical assessment. Specific consistencies, volumes, or textures that emerge as problematic or uncertain during the clinical evaluation should be prioritized during instrumental testing. For this reason, clinical assessment should ideally precede instrumental evaluation, to clearly define its objectives and tailor it to the individual case [58]. The availability of a prior clinical assessment also serves to guide the choice of starting bolus consistency and the “appropriate safety conditions” for the subsequent instrumental exam. Protocols in the literature differ on this point, reflecting variability in clinical and research practice [52]. Some protocols begin testing with thicker consistencies (e.g., IDDSI level 3–4) [59], which are generally associated with a lower initial risk of aspiration but may leave residue that interferes with the interpretation of subsequent thin liquid trials. Conversely, others begin with thin liquids [60], avoiding this interference but carrying a higher initial aspiration risk in patients with more severe impairment. The preceding clinical assessment allows the clinician to identify, for the individual patient, which of these approaches minimizes risk given their specific presentation and risk factors.

The use of a shared and standardized terminology for describing food textures and liquid consistency in clinical and instrumental assessment is pivotal to providing pragmatic and individualized recommendations. The International Dysphagia Diet Standardisation Initiative (IDDSI) framework is a globally recognized classification of food textures and liquid consistencies using defined levels and objective testing methods to support safe and effective OD management [61]. This consensus stresses the professional responsibility of verifying the IDDSI level of any food or liquid used during assessment. It also highlights the importance of clearly reporting the IDDSI levels and volumes of tested foods and liquids in clinical documentation to support the formulation of dietary recommendations that ensure optimal swallowing safety and efficiency. In this context, “optimal safety and efficiency” refers to the balance that best minimizes the risk of penetration and aspiration while also minimizing pharyngeal residue, individualized according to the patient’s specific clinical presentation and risk factors for swallowing-related complications.

Finally, OD assessment should encompass the evaluation of oral medication intake, beginning with its systematic investigation during history taking. Difficulties in swallowing medications are common among people with OD [62,63] and may require targeted assessment. Interest in this area is increasing, and specific protocols for evaluating medication swallowing during instrumental assessment are emerging [64]. By integrating these medical, nutritional, and practical considerations, the assessment evolves from a mere diagnostic procedure into a comprehensive strategy for patient-centered meal management.

Overall, these considerations highlight the need to individualize swallowing assessment to develop tailored dietary and procedural recommendations. Relying solely on standardized recommendations may not adequately reflect the needs and contexts of individual patients.

##### 4.1.1.2. Mealtime Observation


**DO**

It is suggested to conduct mealtime observations under the most ecologically valid conditions possible, namely in the person’s usual environment, with the posture, foods, and caregiver(s) that typically characterize their mealtime.During mealtime observation, it is suggested to identify environmental and personal factors that act as barriers and facilitators for that specific person.To verify the adherence to and the efficacy of the recommendations, one or more follow-up mealtime observations are suggested.

**DON’T**

During the first mealtime observation, the clinician should not modify the mealtime conditions or provide instructions, except when necessary for the person’s safety.It is suggested not to rely solely on patient-, caregiver-, or parent-reported tools, as they cannot independently provide a comprehensive assessment of feeding skills, swallowing function, and mealtime behavior.


Clinical and instrumental evaluations of swallowing represent fundamental components of OD assessment. However, both clinical and instrumental evaluations are typically conducted over a limited time frame and under controlled conditions, testing only a restricted range of consistencies, volumes, and food or liquid types. As a result, they primarily assess swallowing as a *body function* under test conditions, which provide only partial information on swallowing performance during everyday meals. Within the ICF framework, instead, eating and drinking are considered *activities* of daily living that emerge from the interaction between body functions and contextual factors. Accordingly, swallowing performance during meals is influenced not only by swallowing physiology, but also by a range of personal and environmental factors that may act as facilitators or barriers. Moreover, eating and drinking involve multiple interacting domains, including motor abilities (e.g., utensil use and hand-to-mouth coordination), postural control, sensory processing, cognitive functions (e.g., recognizing the mealtime context and sequencing actions), and behavioral regulation (e.g., pacing and inhibition of unsafe behaviors) [29,30]. In this context, mealtime observation provides a critical complement to clinical and instrumental evaluations by enabling the assessment of swallowing performance in an ecological setting [51]. It allows clinicians (1) to determine how safe and efficient swallowing is during everyday meals; (2) to identify discrepancies between what people are able to do under test conditions and what they actually do in daily life; (3) to review and, when necessary, refine diet recommendations and compensatory strategies derived from clinical and instrumental assessments; and (4) to identify modifiable factors that may be targeted through individualized interventions aimed at improving mealtime safety and efficiency.

Mealtime observation is of particular clinical importance in specific patient populations, such as pediatric feeding disorders [64]. It may also be of particular clinical value in at least two situations. First, when there is a discrepancy between clinical and/or instrumental findings and reports from patients or caregivers regarding mealtime experience at home, direct observation during mealtimes may be warranted to assess how eating and swallowing function in daily life. This is particularly important because subjective reports do not always align with observed performance [65]. Mealtime observation can help determine whether this discrepancy reflects limited awareness or insight of the patient or caregiver, or whether it is related to the influence of personal and environmental factors, as well as the interaction with other functions involved in eating. Second, mealtime observation is particularly relevant when the underlying condition suggests that additional factors, such as fatigability, cognitive impairment or response to pharmacological treatment, may negatively affect swallowing safety and efficiency during meals [51].

Currently, there is limited guidance in the literature on how to conduct mealtime observation. While some research protocols and assessment tools employ standardized meals, most studies rely on the patient’s usual meal [66]. Given that the primary aim is to capture real-world functioning, this consensus supports conducting observations under ecological conditions, including the use of typical foods and beverages, habitual posture, usual utensils, and the presence of caregivers who are routinely involved in mealtimes. Similarly, when conducting mealtime observation for assessment purposes, typically during the first observation, clinicians should adopt a primarily observational role. Avoiding modifications to the mealtime context and refraining from providing immediate instructions minimizes observer-induced bias and allows the identification of habitual strategies and behaviors.

In addition to initial assessment, follow-up mealtime observations play a relevant role in evaluating the implementation of recommendations and adaptive coping strategies. Specifically, they allow clinicians to verify both adherence and real-world applicability of prescriptions, in light of individual and environmental resources (e.g., skills, time, financial constraints, caregiver support, cognitive abilities). This is particularly important considering that adherence to OD management recommendations is typically suboptimal [35,36]. Follow-up observations also enable assessment of how effectively recommended strategies are implemented in daily practice. Although repeated in-person observations may not be feasible in all clinical settings, telemedicine may offer a viable alternative. Emerging evidence suggests that swallowing assessment and mealtime observation can be reliably conducted via telehealth [67], supporting continuity of care and monitoring over time.

#### 4.1.2. Nutritional Assessment


**DO**

Malnutrition screening should be performed in all patients with OD using validated tools specific to the clinical setting, age group, and population.It is suggested to diagnose malnutrition through multidimensional assessment tools.Nutritional management of OD must ensure both appropriate texture/consistency and adequate energy-protein and fluid intake.It is suggested to consider the food preferences and cognitive abilities of people with OD to promote adequate intake of the prescribed diet and maximize its acceptability.

**DON’T**

It is suggested not to infer energy intake from mealtime observation alone.It is suggested not to base the nutritional and adiposity assessment of a child with neurological impairment solely on weight and height measurements.It is suggested not to skip periodic nutritional assessments: failure to revise diet prescription according to swallowing function and nutritional status, as well as failure to collect periodic food dietary records, may hinder progress and compromise the safety of dietary therapy.


Malnutrition is frequently associated with OD across the lifespan, with a bidirectional relationship between the two conditions [68]. On the one hand, malnutrition may result from OD due to reduced oral intake secondary to swallowing difficulties [4]. On the other hand, OD itself may be influenced or even caused by malnutrition, as observed in sarcopenic dysphagia [69]. Early identification of malnutrition, or even of its risk, therefore has direct implications for dietary prescription and overall clinical management in people with OD.

Importantly, the impact of OD on nutritional intake cannot be reliably inferred from swallowing assessment alone. There is no direct relationship between the severity of impairment in swallowing and feeding skills and actual oral intake [70]. For this reason, a dedicated pathway for the early identification of people at risk of malnutrition should be implemented. This requires close collaboration between the dysphagia team and the nutrition team (e.g., dietitians and physicians specialized in clinical nutrition with expertise in OD), ensuring an integrated and individualized approach.

The first step in this pathway is nutritional risk screening, which is suggested for all people with OD. Screening should be performed using validated tools appropriate for the specific population and clinical setting. In adults, the European Society for Clinical Nutrition and Metabolism (ESPEN) [71] recommends the use of the Mini Nutritional Assessment Short Form in older adults [72], Nutritional Risk Screening 2002 [73] in hospitalized patients, and the Malnutrition Universal Screening Tool [74] primarily in community and long-term care settings. In pediatric populations, no single tool is universally recommended [75,76]; however, several validated instruments are available, including STRONGkids [77] and STAMP [78].

In people identified as at risk, a comprehensive and multidimensional nutritional assessment should be undertaken [79,80]. In adults, the diagnosis of malnutrition is commonly based on the Global Leadership Initiative on Malnutrition (GLIM) criteria, which integrate phenotypic and etiologic components [80]. In children, evaluation should consider multiple indicators, including clinical signs of undernutrition, growth parameters, body composition proxies, and evidence of poor growth or failure to thrive [79]. Crucially, this assessment must explicitly include the evaluation of hydration status and fluid intake, given the increased risk of dehydration in people with OD [8].

Overall, the nutritional management of OD requires balancing two key objectives: ensuring the selection of food textures and liquid consistencies that promote safe and efficient swallowing (“appropriate texture/consistency”) and maintaining adequate and balanced nutritional and fluid intake. This underscores the importance of close collaboration between the swallowing and nutrition teams in the development of individualized dietary recommendations. Particular attention should be given to people requiring texture-modified diets or thickened liquids. While these strategies are often effective in reducing penetration and aspiration [81,82], they have been associated with reduced nutritional and fluid intake [83,84]. Recent reviews suggest that people receiving texture-modified diets frequently have lower nutritional intake compared with those on regular diets, with implications for malnutrition risk and clinical outcomes [85,86]. Thickened liquids are associated with a progressive decrease in both palatability and fluid intake as viscosity increases [82,87]. Therefore, although they may enhance swallowing safety, their prescription should consider not only immediate effects but also long-term effects [57]. Clinical decision-making regarding the prescription of texture-modified diets and/or thickening fluids should consider: (1) the availability of alternative strategies to ensure safe oral intake of foods and beverages (e.g., use of compensatory postures, maneuvers, and procedures); (2) individual risk factors for pulmonary and nutritional complications; (3) the underlying condition and disease stage (e.g., there is no evidence supporting dietary modifications in people with dementia [88]); and (4) patient preferences. Moreover, dietary modifications should be limited to those strictly necessary. For example, minimum levels of liquid thickening may be sufficient to ensure safe swallowing [82], and the exclusion of mixed consistencies is not always required, particularly when liquid swallowing is preserved. The literature suggests that the swallowing safety of mixed consistencies may largely depend on the swallowing safety of the liquid component [89]. Such considerations may help maximize the benefits of dietary modifications, minimize adverse effects, enhance acceptability in daily life, and reduce the impact on quality of life [90].

Finally, regular follow-up of both nutritional status and swallowing function is essential [79,91]. Periodic reassessment enables clinicians to monitor the adequacy of dietary prescriptions in relation to changes in swallowing function, feeding abilities, and nutritional needs across the lifespan and throughout disease progression or recovery. This continuous reassessment is vital to prevent dietary stagnation and the unnecessary prolongation of restrictive diets, thereby preserving both nutritional health and the patient’s quality of life.

### 4.2. Do’s and Don’ts on Meal Preparation

#### 4.2.1. Beverages, Foods, and Oral Medications


**DO**

When controlling viscosity, it is important to consider the temperature at which the food or beverage will be consumed, as a decrease in temperature may lead to thickening.When using starch-containing beverages and starch-based thickeners, it is suggested to consider that viscosity may be significantly reduced by salivary amylase activity.To increase liquid viscosity in adults, the use of xanthan-gum-based thickeners or other amylase-resistant formulations is suggested.To test the texture and consistency of foods and beverages suitable for the person with OD, it is suggested to use the IDDSI framework and terminology.Chewing should be preserved whenever solid foods can be consumed safely, even if only for hedonic purposes.Before manipulating solid dosage forms in a person with OD, and always following a medical prescription, it is suggested to evaluate whether an equivalent commercially available medication with marketing authorization can be prescribed in an alternative formulation or administered via a different route.

**DON’T**

Do not split tablets into fractions smaller than one-quarter unless explicitly permitted by the manufacturer.


When thickened liquids are prescribed, key aspects of their preparation should be carefully considered and addressed through appropriate education and training of patients and caregivers.

The choice of thickening agent is central. Available products are broadly classified as starch- or gum-based. Starch-based thickeners are widely accessible but also susceptible to salivary amylase, which can rapidly degrade starch during oral processing and reduce viscosity before swallowing, potentially limiting their therapeutic effect [92]. In contrast, gum-based thickeners, such as xanthan gum, or other amylase-resistant formulations, provide greater viscosity stability across conditions and during oral processing, resulting in more predictable bolus flow [93]. Gum-based thickeners were more effective than starch-based thickeners in reducing aspiration [94]. Their shear-thinning properties may further facilitate bolus control without substantially increasing swallowing effort and pharyngeal residue [95,96]. Nevertheless, selection should remain individualized, considering patient tolerance and perceived palatability and acceptability.

In pediatric populations, additional caution is required due to physiological and developmental considerations. Although thickening may improve swallowing safety [97], potential risks related to hydration, nutritional adequacy, fatiguability and pulmonary risk, particularly in infants, should be considered [98]. The choice of thickener is also critical: starch-based agents may lose viscosity due to amylases, whereas gum-based products, despite greater stability, have raised safety concerns in vulnerable groups such as preterm infants, including reports of gastrointestinal complications [99,100]. Consequently, the use of commercial thickeners in newborns and young infants remains controversial. Candidates for this treatment should be carefully selected by balancing potential risks against benefits in the discussion with the family and care team [98,99,100,101]. Regular reassessment is suggested to minimize duration and avoid unnecessary prolongation, and alternative strategies (e.g., positioning, flow modification, pacing) should be considered within a multidisciplinary approach.

Temperature and time are also relevant factors which influence the rheological properties of thickened liquids. Lower temperatures are generally associated with increased viscosity, particularly in starch-based preparations, meaning that viscosity at consumption may differ from that at preparation [93]. Viscosity may change over time depending on the commercial formulation, with some products exhibiting a decrease and others a progressive increase in viscosity [95]. This variability may affect swallowing safety and efficiency. Therefore, viscosity targets should reflect actual consumption conditions, especially in settings where delays between preparation and intake occur. A practical strategy, at least for beverages, is to prepare small volumes that can be consumed promptly.

Standardized frameworks such as IDDSI [61] provide practical guidance for classifying the consistency of thickened liquids and texture-modified foods, while accounting for factors such as temperature-related variability that may influence final consistency, although the effect of saliva on bolus consistency during oral processing is not considered within the framework. The lack of standardized terminology and consistency control procedures may lead to discrepancies between prescribed and prepared textures, resulting in patients receiving foods that require more complex swallowing abilities than clinically intended, potentially increasing choking risk and reducing oral intake [102]. The IDDSI testing methods are standardized, low-cost, and easy-to-perform procedures that allow verification of food and liquid consistency at serving, thereby supporting reproducibility across settings, from clinical environments to home care and residential or rehabilitation facilities. Adequate training of caregivers and people with OD is essential to ensure correct application of these methods, including testing procedures and preparation techniques required to achieve the target IDDSI level.

Importantly, IDDSI also enables a more nuanced approach to texture and consistency modification. The availability of multiple levels allows clinicians to move beyond the routine prescription of highly restrictive consistencies (e.g., Level 4) for all patients, facilitating more individualized and graded adjustments between Levels 4 and 7. Avoiding unnecessary restrictions may help preserve eating-related quality of life [90] and support active use of swallowing function during daily meals, in line with principles of neuroplasticity [103]. In this context, preserving chewing should be considered a relevant goal whenever solid foods can be safely consumed, even when intake is primarily for hedonic purposes. Chewing contributes not only to bolus preparation but also to nutritional status, oral function, and eating-related quality of life. Evidence from a recent systematic review indicates that mastication influences bolus formation, salivary secretion, and swallowing efficiency, while also affecting gastrointestinal function and nutrient bioavailability [104]. Conversely, impaired chewing has been associated with poorer nutritional outcomes, as reduced masticatory function may limit dietary variety and lead to avoidance of nutrient-dense solid foods [105]. Within this framework, IDDSI Levels 6 (soft and bite-sized) and 7EC (regular, easy-to-chew) may support the preparation of foods that maintain masticatory function even in people with reduced chewing ability, while preserving swallowing safety.

Medication management is a critical yet often overlooked aspect of care in people with OD [106,107]. Difficulty in swallowing solid dosage forms frequently leads to medication manipulation (e.g., crushing tablets or opening capsules), which may facilitate administration but can introduce risks, including altered pharmacokinetics, dose inaccuracy, and reduced efficacy, particularly for modified-release or enteric-coated formulations [108,109,110]. Before manipulating solid dosage forms, it is essential to consider the availability of therapeutically equivalent, authorized alternatives (e.g., liquid formulations, dispersible tablets, or alternative routes of administration) [111,112], which ensure dose accuracy and preserve pharmacological properties. When manipulation is unavoidable, it should follow manufacturer’s instructions and pharmacy guidance. Tablets should not be split into fractions smaller than one quarter unless explicitly permitted, as further subdivision may compromise dose uniformity and stability [113]. Crushing or splitting may also affect palatability and adherence [114]. In the Italian context, the manipulation of solid oral dosage forms should be performed only under medical prescription and in accordance with the national safety recommendations [112]. Preliminary evidence from few available in-vivo studies suggests that co-administration with thickened liquids may alter drug disintegration and dissolution, potentially slowing drug release and reducing bioavailability [115]. These effects vary depending on the drug formulation and on the type and the amount of thickening agent used [115]. Medication management in OD therefore requires a multidisciplinary approach. Education of patients and caregivers, along with regular medication review, is essential to ensure safe administration and minimize unnecessary manipulation.

#### 4.2.2. Mealtime Environment


**DO**

Environmental (physical, social, cultural) factors should be considered to identify intervention areas aimed at improving mealtime management.When adequate, well-fitted, and accepted by the person, the use of dental prostheses during eating is suggested.It is suggested to support the person’s ability to self-feed, including through the use of assistive devices, to promote a sense of competence and maintain self-esteem.If the person can safely maintain a seated position, with or without assistive devices, it is appropriate to verify and, if necessary, adjust posture before the meal so that they sit on the ischial tuberosities, the trunk is stable, and the feet are fully supported.In children with OD, an upright or slightly reclined trunk posture is suggested to improve feeding safety and efficiency, although this should be on a case-by-case basis.A child who has not yet developed functional sitting or who presents tone abnormalities (spasticity or hypotonia) must be well supported in the highchair or seating system during meals.

**DON’T**

Do not feed a person with OD before checking whether head and neck posture interferes with meal safety and efficiency and, if necessary, appropriately adjusting it.It is suggested not to feed a child with poor trunk control in an unsupported upright position, as this may limit the development of an adequate chewing pattern and increase aspiration risk.It is suggested to limit the use of distractions, such as television, during mealtimes, after verifying that they hinder safety and efficiency.


Mealtime management in OD extends beyond food and liquid modification and should incorporate environmental optimization, considering physical, social, and cultural factors that influence eating performance. These dimensions are consistent with biopsychosocial models such as the ICF and occupational therapy frameworks (e.g., Person–Environment–Occupation model), which emphasize the interaction between individual abilities, task demands, and environmental context in shaping participation during meals [28,116,117]. Accordingly, environmental factors should be systematically assessed to identify modifiable determinants of safe, efficient, and enjoyable eating. An integrative review applying the Five Aspects Meal Model (room, meeting, product, management control system, and atmosphere) to care home mealtime interventions found that even when targeting a single environmental aspect, outcomes often extend across multiple domains, suggesting that different aspects of the mealtime environment can work synergistically to promote effective mealtimes [118]. This section focuses on the physical environment.

Among physical factors, dentition status and the presence of functioning dental prostheses are particularly relevant. It is documented that a low number of eruptive teeth negatively impacts masticatory performance [119]. The use of well-fitted dental prostheses during meals may improve masticatory efficiency and pharyngeal swallowing function [119,120]. Additionally, adequate dentures may positively influence eating-related quality of life [120,121,122]. Conversely, poorly fitting or uncomfortable prostheses may reduce swallowing efficiency [123] and food intake [124]. For these reasons, the indication to use dental prostheses during meals should be encouraged when accepted by the person with OD.

Postural alignment is a critical aspect for swallowing safety and efficiency. Adequate alignment of the trunk and head supports epiglottic inversion and, consequently, laryngeal vestibule protection [125]. Head posture also influences the timing and extent of hyoid movement, which is critical for both airway protection and upper esophageal sphincter opening; when compromised, it may increase the risk of pharyngeal residue and aspiration [126,127]. Additionally, excessive cervical muscle tone can further alter hyoid bone kinematics [128]. Regarding the oral phase of swallowing, inadequate head posture can negatively affect chewing efficiency and bolus formation by impairing jaw stability and coordination [128], reducing masticatory muscle activation [129], and decreasing intra-oral tongue pressure [130]. Therefore, appropriate alignment of the head, neck, and trunk should be ensured to support both oral motor control and pharyngeal function. Stable sole-ground contact is equally critical. Studies have demonstrated that the absence of sole-ground contact reduces suprahyoid and sternocleidomastoid muscle activity [131], as well as chewing stability and masticatory performance [132]. Findings from the literature translate into specific expert consensus statements for postural management at mealtimes. In adults, when a seated position can be safely maintained, posture should be optimized before the meal, ensuring alignment on the ischial tuberosities, trunk stability, and adequate foot support, using seating and positioning devices as needed. Head and neck alignment should always be verified and, if necessary, corrected prior to feeding, as suboptimal positioning may impair bolus control and airway protection. “Appropriately adjusting posture” in this context means approximating, as closely as the patient’s clinical and motor condition allows, a neutral head-neck alignment or a slightly forward-flexed (chin-tuck) position, both of which support airway protection during swallowing. In pediatric populations, positioning requires particular attention due to developmental factors. An upright or slightly reclined trunk position may improve feeding safety and efficiency, although it should be individualized [133]. Children without functional sitting or with tone abnormalities (e.g., spasticity or hypotonia) require appropriate support through highchairs or specialized seating systems to ensure stability during meals. Conversely, unsupported upright positioning in children with poor trunk control should be avoided, as it may impair oral motor development and increase aspiration risk [134].

Supporting the person’s ability to self-feed is another key determinant of mealtime experience. Promoting autonomy through suitable positioning and the use of assistive devices, when appropriate, may enhance participation, reinforce a sense of competence, and maintain self-esteem [135,136,137,138,139]. Qualitative evidence highlights that mealtime experiences in people with disabilities are closely linked to dignity, identity, and social participation, further supporting the importance of preserving active involvement in feeding [136,137].

Finally, environmental modulation should also consider attentional and contextual factors. While minimizing distractions (e.g., television) may improve safety and efficiency in some people, this should be evaluated on a case-by-case basis. A systematic review and meta-analysis highlighted that watching television while eating increases food intake through reduced attention to internal satiety cues, an effect that may be undesirable in people at nutritional risk [140]. However, in the context of dysphagia, reduced attentional engagement with food and reduced self-monitoring of swallowing safety are additional concerns. Nursing assistants with direct mealtime experience report that distractions such as noise and crowding can significantly inhibit residents’ food intake, and that the benefit of playing music in the dining area depends on the individual resident, reinforcing the importance of an individualized approach [141]. In pediatric populations, particularly in children with pediatric feeding disorders (PFD), the use of distractions may sometimes be necessary to ensure adequate oral intake. However, they should be carefully monitored and limited whenever possible, privileging less absorbing or less intrusive stimuli to minimize interference with feeding skills development and swallowing safety. Current conceptual and clinical frameworks for PFD emphasize the importance of responsive feeding approaches and caution against excessive reliance on external distractors during meals [142].

Beyond distraction control, the broader physical and social mealtime environment constitutes a meaningful and modifiable intervention target, particularly for older adults in institutional settings. The structure, furniture, organization and accessibility of dining rooms in long-term care facilities are increasingly recognized as an important catalyst for implementing a culture based on person-centered care philosophy, and evidence suggests that purposeful environmental modifications can yield measurable benefits [143]. In a qualitative study, the effects of structural changes in the dining room of a long-term care facility included supporting independence and autonomy, creating familiarity and mealtime enjoyment, and providing a place for social experience [144]. These results highlight the integral role of the built environment alongside organizational support in providing person-centered mealtime care. A systematic review of communal dining and dining room enhancement interventions across acute, sub-acute, rehabilitation, and aged-care settings found that communal dining was associated with greater energy and protein intake and higher quality of life measures compared to non-communal mealtime settings, while dining room enhancement interventions (including changes to décor, lighting, table settings, and ambient music) overall contributed to increased intake of food, energy, protein, and fluid [145].

### 4.3. Do’s and Don’ts on Mealtime Experience in Daily Life

#### 4.3.1. Psychosocial Implications of OD


**DO**

In assessing and managing the implications of swallowing disorders, it is necessary to consider the entire family and social context (family-centered care).

**DON’T**

In people with OD, the social, symbolic, and psychological aspects of eating as an activity of daily living should not be overlooked.Do not infer the nature and extent of the family and social implications of OD solely from the severity and pathophysiology of the swallowing disorder.


Eating has not only a nutritional function but is also a meaningful activity embedded within social, cultural, and relational contexts. The onset of OD entails substantial changes in mealtimes that extend beyond physiological impairment, affecting identity, social participation, and emotional well-being [146]. Qualitative and mixed-methods studies have documented experiences of embarrassment, anxiety, and social withdrawal, as well as reduced enjoyment of eating [147,148,149], highlighting the need to address psychosocial dimensions alongside clinical management. Importantly, the social, symbolic, and psychological meanings of eating should not be overlooked. Food is closely linked to cultural identity, habits, and personal preferences, and restrictive interventions may unintentionally affect these dimensions [150,151]. Person-centered approaches that incorporate individual values and lived experiences have been advocated to balance safety with quality of life and participation [22,24,152].

A particularly relevant dimension in this regard is the impact of OD on social eating—that is, the ability to eat and drink in the company of others. People with OD frequently report difficulties in sharing meals, with consequences for social participation, relational dynamics, and quality of life. A recent clinical practice statement provided a comprehensive overview of social eating challenges in people with cancer and proposed practical guidance for their clinical management [153]. Although developed within an oncology framework, its core recommendations are broadly applicable to any condition affecting swallowing and eating function, including OD. Two simple filter questions were proposed to identify patients at risk: “How has your swallowing difficulty changed your eating habits with other people, whether in a restaurant, at home, or at a social occasion?” and “How has it affected your drinking behavior in social settings?” [153]. Given the limited attention this dimension has received in the context of OD, systematically incorporating such screening into routine clinical assessment represents a feasible and meaningful step toward addressing social eating challenges, which may represent a key determinant of participation and quality of life.

Within this perspective, assessment and intervention should adopt a family- and context-centered approach, involving caregivers and the patient’s broader social environment. The impact of dysphagia frequently extends to family members, influencing mealtime routines, roles, and interpersonal dynamics. Systematic reviews found that the presence of OD consistently increases burden among family caregivers [21,154]. A qualitative meta-synthesis further documented that caregivers undergo considerable lifestyle changes alongside physical and emotional distress, and they consistently perceive insufficient professional, educational, and psychological support [20]. Crucially, the management of dysphagia itself (including the implementation of dietary modifications, mealtime compensations, postural adjustments, and alternative feeding) constitutes a major source of time and psychological cost for caregivers [21,154]. Therefore, family experiences and caregiver burden are integral components of OD management and should be explicitly considered in care planning.

Furthermore, the impact of oropharyngeal dysphagia on daily life cannot be inferred solely from the severity or pathophysiology of the swallowing disorder. People with similar levels of impairment may experience markedly different psychosocial and emotional consequences, depending on personal resources, expectations, coping strategies, and the availability of social and environmental support [24,149,155]. Importantly, significant psychosocial impact has been documented even in the absence of severely abnormal swallowing findings, highlighting the limited predictive value of physiological measures alone for the person’s lived experience [155]. These factors shape how the condition is perceived and managed in everyday life, influencing participation, adaptation, and quality of life. This variability underscores the need for a comprehensive, individualized assessment that extends beyond swallowing function to include the person’s lived experience and contextual factors [22,24,146,156].

#### 4.3.2. Caregiver–Child Interaction and Feeding Styles


**DO**

It is suggested to frequently share family meals, emphasizing the quality of interaction and allowing the child to observe others consuming healthy foods.Caregivers should be encouraged to adopt a responsive feeding style that promotes the child’s self-regulation and is associated with healthy eating habits.

**DON’T**

It is suggested that caregivers of children with OD should avoid authoritarian, indulgent, or uninvolved feeding styles.


Feeding in childhood is a relational process in which caregiver–child interaction plays a central role in shaping eating behaviors, skills development, and mealtime experiences. Beyond swallowing safety, the quality of interpersonal exchanges during meals influences the child’s engagement, learning, and emotional response to feeding. This is particularly relevant in children with PFD, where feeding difficulties arise from the interaction of medical, nutritional, feeding skill, and psychosocial domains [157].

This expert consensus suggests sharing family meals “frequently,” which means doing so as often as feasible within the family’s organizational and logistical constraints. Sharing family meals represents an important opportunity to support feeding development. Observational learning allows children to model eating behaviors and food acceptance, while repeated exposure and social context contribute to shaping their food preferences and reducing neophobia [158,159,160]. Eating habits are also influenced by transgenerational processes, whereby caregivers’ attitudes, practices, and cultural norms are transmitted across generations [158]. These mechanisms highlight the importance of the family environment in promoting healthy and adaptive eating behaviors.

Within this context, a responsive feeding style is advisable [142]. Responsive feeding involves recognizing and appropriately responding to the child’s hunger and satiety cues while providing structured guidance. This approach has been associated with improved self-regulation, healthier eating patterns, and better nutritional outcomes, and is considered a key component in the management of PFD [157,161]. In addition, responsive feeding may reduce mealtime stress and support more positive caregiver–child interactions. Position statements further emphasize that supportive, non-coercive feeding environments are essential for fostering children’s autonomy and long-term healthy eating behaviors [161].

Conversely, non-responsive feeding styles—such as authoritarian, indulgent, or uninvolved approaches—may negatively affect feeding development. These patterns may interfere with the child’s ability to regulate intake, increase mealtime conflict, and contribute to maladaptive eating behaviors [158,159,160,161]. In children with PFD, such dynamics may further complicate feeding management by increasing resistance and anxiety, or reducing cooperation during meals [157,162,163].

Overall, pediatric OD management should integrate relational and contextual aspects of feeding, supporting caregivers in adopting responsive, structured, and developmentally appropriate practices within a comprehensive, multidisciplinary framework [157].

#### 4.3.3. Sensory and Hedonic Dimensions of Eating


**DO**

The diet of older adults must have qualities that positively influence their quality of life. Foods should be prepared with high-quality ingredients, in ways consistent with the diners’ traditions, and presented in a hygienically safe and aesthetically pleasant setting.Foods offered to older adults should stimulate multisensory perception.In people with total artificial nutrition, it is suggested to verify whether small amounts of food can be safely consumed for hedonic purposes, while respecting personal preferences and tastes.


Mealtime management in OD should not be limited to physiological safety but should also address the sensory and hedonic dimensions of eating. This perspective is particularly relevant in older adults, whose food intake is closely linked to quality of life, social participation, and overall well-being [164]. Accordingly, preserving the meaning of eating beyond its purely nutritional function is essential. Standardized or overly restrictive diets, although sometimes necessary to reduce aspiration risk, may lead to reduced appetite, poor adherence, and an increased risk of malnutrition [85,86]. Moreover, considering that eating behaviors and food-related practices are shaped by social and cultural factors, aligning food preparation with cultural and culinary traditions and individual habits and preferences may optimize appetite and meal intake [149]. The quality of the ingredients themselves is equally relevant: whether used fresh or industrially processed for texture modification, ingredients should be derived from good-quality source products and selected for their nutritional value and palatability.

The role of multisensory stimulation is also central. Aging and neurological conditions are frequently associated with sensory decline, including reduced taste and smell perception [164]. Flavor perception is inherently multisensory, resulting from complex interactions between gustatory, olfactory, and trigeminal inputs, as well as visual and contextual cues [165]. Enhancing visual appeal, aroma, flavor intensity, and texture variability may therefore help compensate for sensory changes and promote food intake [164], while improving the eating experience and maintaining oral-motor stimulation. In this regard, emerging evidence from studies conducted in long-term care settings suggests that improving the visual and sensory characteristics of texture-modified foods (e.g., through molding or reshaping their appearance to resemble the original one) may increase food intake and improve mealtime satisfaction compared with traditional pureed diets [166,167]. This is particularly important when texture-modified diets are prescribed, as these are often perceived as monotonous or unappealing and are associated with reduced energy and protein intake [85,86]. Neuroimaging studies have demonstrated that texture-modified foods with realistic, original-like appearance, as opposed to unstructured purées, elicit significantly greater activation of the visual cortex and reward-related areas, as well as higher subjective appetite ratings, both in healthy individuals and in people with dysphagia [168,169]. This evidence suggests that the visual unrecognizability of typical pureed food may itself contribute to reduced appetite and intake, independently of palatability. At the same time, the pursuit of sensory enhancement should remain compatible with safety requirements and prescribed texture levels.

Finally, the consensus highlighted the potential role of hedonic oral intake in people receiving total artificial nutrition. Although usually discouraged due to safety concerns, emerging literature on “Eating and Drinking with Acknowledged Risk” and “comfort feeding” supports a more individualized and ethically grounded approach, in which limited oral intake may be allowed to preserve pleasure, dignity, and quality of life, particularly in advanced disease or palliative contexts [170,171]. This approach requires careful assessment of swallowing safety, explicit risk–benefit discussion, and shared decision-making with people with OD and their caregivers, as well as clear documentation and interprofessional agreement.

### 4.4. Do’s and Don’ts on Patient and Caregiver Education

#### 4.4.1. Patient-Centered Education and Interprofessional Collaboration


**DO**

In quality improvement processes and when optimizing health outcomes, it is appropriate to involve the person with OD, their family, and the community.When educating the person with OD and their informal caregivers, it is suggested to verify learning by asking them to restate information in their own words and provide practical examples, using communication modalities appropriate to the person’s cognitive and linguistic abilities.When educating the person with OD and their informal caregivers, it is suggested to complement written informational materials with video resources, oral explanations, and in-person demonstrations, while avoiding providing excessive information in advance or concentrated in a single session.Developing interprofessional collaboration can improve outcomes in OD care, as well as the work environment, staff well-being, and organizational functioning.

**DON’T**

It is suggested not to use highly specialized scientific terminology without simple explanations, and not to overlook the health literacy level of the person with OD and their caregiver.To ensure appropriate care delivery, it is suggested to avoid authority imbalances and friction related to “professional boundaries” that may arise from limited understanding of each other’s roles and responsibilities.


Beyond the implementation of specific clinical interventions, effective care requires the active involvement of people with OD, their families, and, when appropriate, the broader social context. Engaging patients and caregivers in decision-making and care processes has been associated with improved adherence, better health outcomes, and greater satisfaction with care [172,173].

Education of patients and informal caregivers is a key aspect of mealtime management. The educational needs of people with OD and their informal caregivers are multidimensional and extend beyond the mere transmission of information [22,174]. Informational needs include the provision of clear, tailored explanations to support understanding of the rationale underlying dietary recommendations and procedural strategies, which are essential to promote adherence and engagement [174,175]. In parallel, practical needs involve training in specific skills required for daily management, such as appropriate food and liquid preparation, safe feeding techniques, and the implementation of oral care. Tailoring communication to the individual’s cognitive and linguistic abilities, as well as to their level of health literacy, while avoiding the use of overly technical language, is essential to ensure effective knowledge transfer [176,177]. Accordingly, educational strategies should be selected based on the specific objectives of the intervention. Approaches that actively verify understanding can support comprehension, retention of information, and the correct application of recommendations in daily life [178]. Examples include the teach-back method, in which people are asked to restate information in their own words, and the “show-me” approach, which involves demonstrating a task or skill [178]. Furthermore, educational interventions should address not only safety and nutritional management, but also autonomy, coping strategies, and caregiver involvement. Combining different educational modalities (e.g., written materials, verbal explanations, video resources, and hands-on demonstrations) may enhance learning outcomes compared with single-modality approaches [179]. At the same time, avoiding information overload, particularly in the early stages of care, appears important to facilitate comprehension and engagement, thereby supporting patient empowerment and active participation in care.

Interprofessional collaboration also plays a crucial role in supporting patient empowerment through education. Given the complexity of OD, which often involves medical, nutritional, functional, and psychosocial dimensions, coordinated input from multiple professionals is required. Consistent and aligned communication across professionals is essential to ensure that educational messages are coherent, comprehensible, and applicable in daily life. Accordingly, reducing hierarchical interaction patterns and communication barriers, both between professionals and patients and among professionals, is critical. Evidence indicates that effective interprofessional teamwork is associated with improved clinical outcomes, enhanced patient safety, and greater efficiency of care delivery [180,181]. Recent evidence further highlights that interprofessional collaboration is influenced by multiple factors, including shared goals, role clarity, communication quality, collaborative leadership, and organizational support, and it may also positively impact staff well-being and job satisfaction [182,183].

#### 4.4.2. Reduction of Pulmonary Risk


**DO**

In patients who present episodes of silent aspiration, it is suggested to assess the effectiveness of protective maneuvers (coughing, forced expiration) during meals and investigate potential causes of ineffectiveness.It is suggested to verify and ensure adequate oral hygiene before eating.Assessment of oral health status should preferably be performed by healthcare professionals using validated scales, while considering the clinical condition of the person and documenting the results in the clinical record.Oral health status must be assessed periodically in all people with known or potential alterations of the oral cavity.When compatible with the patient’s clinical condition, the use of an electric toothbrush is preferable, as it is more effective at reducing plaque and biofilm compared with a manual toothbrush.Accurate daily oral hygiene at home is suggested to reduce the risk of bacterial colonization and pulmonary complications.Oral health and cleanliness of teeth, periodontal tissues, and oral mucosa must be maintained, as these factors facilitate bolus perception and processing during the oral preparatory phase.The Free Water Protocol may be used in selected cases, always in conjunction with meticulous oral hygiene and exclusively in settings that ensure strict patient adherence.

**DON’T**

For adequate oral hygiene, the use of gels or mouthwashes alone is insufficient without the mechanical action of toothbrushing.Do not use chlorhexidine-based mouthwashes for daily use, except in cases of gingivitis or periodontitis.


Patient and caregiver education represents a key component of risk mitigation in OD. Emphasis should be placed on strategies aimed at reducing pulmonary complications, including airway protection techniques and oral hygiene practices, which require not only awareness but also practical training to be effectively implemented in daily life. Indeed, aspiration is a necessary but not sufficient condition for the development of aspiration pneumonia. According to the BOLUS framework, the likelihood of an adverse event from aspiration is modulated by several factors that were grouped into five categories: bolus variables, oral care, lifestyle and activities of daily living, unintended/iatrogenic risks, and system health [184,185]. Identifying and targeting modifiable factors can mitigate the risk of pulmonary complications. Accordingly, the present statements emphasize the importance of airway protection and oral health as integral components of mealtime management in OD.

Airway protection mechanisms represent a critical aspect of dysphagia management, particularly in people with silent aspiration [186]. In such cases, evaluating the effectiveness of protective responses, such as voluntary or reflexive coughing and forced expiration, may help identify patients at higher risk and guide clinical decision-making. Voluntary and reflex cough tests have shown discriminative abilities to detect penetration and aspiration in several populations at risk for OD [187,188,189,190]. Additionally, voluntary and reflexive peak cough flow were reported to be independent predictors of pneumonia in people with OD [191,192,193]. When clinically indicated, instrumental assessment may be required to verify the effectiveness of airway protection strategies, since bedside observation alone may fail to detect ineffective airway protection, particularly in cases of silent aspiration [194]. Furthermore, patients and caregivers should receive specific training not only on how to apply airway protection strategies correctly, but also on when they should be applied and how to recognize whether the response has been effective or ineffective in daily practice.

Poor oral hygiene is associated with increased oral bacterial colonization, which may contribute to the development of aspiration pneumonia, particularly in vulnerable populations such as older adults and people with neurological disorders [195,196,197]. A meta-analysis conducted during the development of recommendations for post-stroke dysphagia management by the European Stroke Organisation and the European Society for Swallowing Disorders found that oral health care interventions were associated with a trend towards reduction of pneumonia development and a significant improvement in oral health, although the level of evidence was low [198]. A systematic review on adults and children with OD instead provided limited evidence that oral health care interventions reduce aspiration pneumonia incidence and improve oral health in people with OD [199]. While the literature on the effects of oral health care interventions in children with OD was found to be scarce, studies in adults indicated that oral cleaning, twice a day, with an antibacterial toothpaste in combination with interdental cleaning and the Free Water Protocol (i.e., a protocol allowing selected people with aspiration for thin liquid to consume thin water between meals and after accurate oral hygiene) proved to be the best intervention to improve oral health [199,200]. Therefore, regular and effective oral health care, including mechanical plaque removal, seems to be a reasonable preventive strategy. In this context, powered toothbrushes have been shown to be more effective than manual toothbrushes in reducing plaque and oral biofilm [201]. Furthermore, the combination of toothbrushing with antiseptic agents appears more effective than antiseptics alone, reinforcing the importance of mechanical cleaning [202]. Accordingly, relying exclusively on mouthwashes without toothbrushing is insufficient to ensure adequate oral hygiene. The routine use of chlorhexidine should also be carefully considered, as its benefits are mainly supported in specific clinical conditions rather than for long-term daily use [203].

Systematic assessment of oral health status is also essential. The use of validated tools, such as the Oral Health Assessment Tool [204] or the Brief Oral Health Status Examination [205], and regular monitoring may facilitate early identification of oral conditions that could negatively affect swallowing function, such as pain, mucosal lesions, or poor dentition. Maintaining the integrity and cleanliness of oral structures supports not only oral health but also the sensory and motor processes involved in bolus preparation and oral phase control [206,207].

The Free Water Protocol represents a specific example of a risk-managed approach [208]. It allows selected people with OD who aspirate thin liquids to consume thin water between meals while maintaining prescribed thickened fluids during meals. Its implementation requires strict oral hygiene, appropriate timing of water intake (at least 30 min after meals), adequate alertness, and careful patient selection to exclude people at high risk of unsafe intake. Although traditionally restricted, the controlled introduction of water intake in selected patients is associated with potential benefits, including improved hydration and quality of life, without a clear increase in adverse events when strict criteria are met [208]. However, its implementation requires not only careful patient selection and rigorous oral hygiene but also structured education and training for patients and caregivers to ensure correct implementation and adherence in everyday settings.

### 4.5. Strengths and Limitations

This study presents several strengths. First, it involved a large and multidisciplinary panel of experts, ensuring broad clinical and academic representation across relevant domains. Second, the Delphi methodology allowed systematic refinement of expert consensus statements and achieved high response and retention rates across rounds. Third, the focus on practical, clinically applicable statements enhances the translational value of the findings for everyday clinical practice.

Despite these strengths, several limitations should be acknowledged. First, although the panel was multidisciplinary and included professionals with expertise in different aspects of dysphagia management, patients and informal caregivers were not directly involved in the consensus process. Their inclusion is a recognized priority, and future research should employ co-design methodologies to capture the lived experience, nutritional priorities, and practical challenges of daily meal management. Nevertheless, the present work specifically aimed at developing clinically oriented statements based on expert practice and professional consensus. Regarding panel composition, it should be considered that the panel was geographically concentrated, with most of the experts (60%) affiliated with institutions in Northern Italy, partially reflecting the origin of this consensus at a course held in Milan. Panel selection prioritized professional expertise, academic standing, and national recognition rather than geographic representativeness. However, regional variations in clinical practice and resource availability across Italy were not systematically addressed and may be a relevant consideration for future initiatives. Furthermore, some statements did not reach consensus, particularly those addressing highly specific populations or contexts, such as nutritional management in the pediatric population with OD. In these cases, a high proportion of “unable to express an opinion” responses was observed, likely reflecting the limited expertise of part of the panel in these more specialized areas. This aligns with panel composition: only 15% of the panel had expertise in nutrition, and only 11% had expertise in neonatal feeding. Similarly, experts in the fields of pharmacy, dental care, and physical rehabilitation were poorly represented, while the majority of the panel consisted of swallowing specialists (SLP, phoniatricians, ENT). Notably, a sensitivity analysis recalculating agreement while excluding “unable to express an opinion” responses from the denominator shows that statements #9, #10, #22, and #28 would reach the 75% agreement threshold, suggesting that these highly specialized areas warrant dedicated, topic-specific guidance. Therefore, although the multiprofessional composition of the panel represented a major strength of this study, it was not sufficient to ensure homogeneous expertise across all specialized areas of dysphagia management. Taken together, the geographic concentration of the panel and the uneven representation of certain professional groups and pediatric specialties indicate that the generalizability of these findings should be considered with caution, particularly regarding their applicability beyond the Italian context and to areas of practice requiring highly specialized expertise not fully represented within the panel.

A major limitation of the study is the absence of a formal systematic review underpinning each statement. The consensus process was primarily designed to address practical aspects of mealtime management that are frequently encountered in clinical practice but are often insufficiently covered by high-quality evidence or existing guidelines. Indeed, several areas addressed in the present work are supported by limited or heterogeneous literature, making expert consensus particularly relevant in supporting clinical decision-making. However, readers should be aware that the majority of the statements were based on a very low level of evidence or on expert opinion only. Indeed, the level of evidence supporting the statements varied considerably across the four domains addressed by this consensus. More extensively studied areas, including oral hygiene and the rheological properties of thickening agents, were supported by a comparatively higher level of evidence, whereas the literature remained sparse or heterogeneous for the other areas. Additionally, statement development did not include a formal assessment of the strength of the recommendations, as expected in GRADE-based guidelines. Consequently, the level of consensus on a statement only reflected the direction of experts’ agreement and should not be interpreted as a measure of the certainty of the underlying evidence or as an indicator of the strength with which each statement should be applied in clinical practice.

Another limitation is that participants received aggregated, rather than individualized, feedback between Delphi rounds. This approach was chosen to provide panel members with an overview of group responses while maintaining the feasibility and manageability of the consensus process. Individualized feedback would have been more closely aligned with the original Delphi methodology and might have further encouraged each expert to critically re-evaluate their own responses in light of the panel’s opinions.

Finally, the response format was based on three response options plus an additional ‘unable to express an opinion’ category. This simplified structure was adopted to facilitate participation and reduce ambiguity in the interpretation of responses. At the same time, it may have limited the ability to capture more nuanced positions compared with broader ordinal agreement scales.

## 5. Conclusions

The present consensus highlights that mealtime management in people with OD should adopt a person-centered and individualized approach, extending beyond standardized dietary prescriptions toward precision-based nutritional care. Dietary recommendations should be tailored to the specific person based on a comprehensive swallowing assessment, integrated with mealtime observation and nutrition-focused evaluation when appropriate. Clinical decision-making should integrate swallowing safety, nutritional needs, patient preferences, and quality of life considerations. Importantly, mealtimes should be understood as complex activities involving physical, social, relational, and cultural dimensions, all of which should be addressed as integral components of care. Moreover, support and recommendations for OD management should not only target people with OD, but also family members and informal caregivers, who frequently face specific educational, practical, and emotional needs related to meal management in daily life. Importantly, providing recommendations alone is insufficient to ensure their effective implementation and long-term adherence. Educational interventions should therefore aim to provide people with OD and their caregivers with the knowledge, skills and practical tools necessary to understand the rationale underlying recommendations, apply them correctly, and evaluate their effectiveness in everyday situations. To achieve these goals, patient and caregiver education should combine multiple educational strategies and modalities, including practical training and active verification of understanding. In this context, interprofessional collaborative practice plays a central role in promoting coherent communication among healthcare professionals and supporting a shared, coordinated, and patient-centered approach to care, in which people with OD and their caregivers are considered integral members of the care team. Consistent with this, mealtime management of OD should be inherently multidisciplinary, highlighting the need to equip institutional teams—and, more broadly, the healthcare system—with all relevant qualified professionals to optimize patient care.

The statements developed through this consensus process reflect the collective judgment of a multidisciplinary expert panel at a given point in time, grounded to varying degrees in the available literature. They document what participating experts consider appropriate practice, not observed clinical behaviors, and are intended to orient clinical practice and to identify priorities for future investigation, rather than to constitute confirmation that every statement improves clinical outcomes. Future research should prioritize the empirical validation of these expert consensus statements, through studies evaluating their implementation, feasibility, and clinical impact across different care settings.

## Figures and Tables

**Table 1:** Characteristics of the panel (N = 53).

Variable	n	%
***Professional background***		
Phoniatrician/ENT	15	28.3%
Speech and language pathologist	15	28.3%
Physician specialized in clinical nutrition	5	9.4%
Dietitian	3	5.7%
Nurse	3	5.7%
Dentist	2	3.8%
Physiotherapist	2	3.8%
Dental hygienist	1	1.9%
Expert in educational sciences	1	1.9%
Food technologist	1	1.9%
Occupational Therapist	1	1.9%
Pharmacist	1	1.9%
Physiatrist	1	1.9%
Psychologist	1	1.9%
Radiologist	1	1.9%
***Role***		
Clinician	34	64.2%
Academic/researcher	19	35.8%
***Sex***		
Male	19	35.8%
Female	34	64.2%
**Years of professional experience** *	22.3 ± 10.3 (5–37)	

* For the variable “Years of professional experience”, the mean ± standard deviation (range) were reported.

**Table 2:** Questions of the Delphi consensus.

**Diet prescription**
How should swallowing function be assessed to inform individualized diet recommendations in people with OD?
How should a nutrition-focused physical examination and comprehensive assessment be integrated to inform individualized diet recommendations in people with OD?
**Meal preparation**
How should foods, beverages, and oral medications be adapted to improve swallowing safety and efficiency in people with OD?
How should the mealtime environment be modified to maximise comfort and support safe, efficient, and participatory eating in people with OD?
**Mealtime experience in daily life**
How should psychosocial, relational, and experiential aspects of eating be addressed to promote social participation and health-related quality of life in people with OD?
**Patient and caregiver education**
How should education on meal management be delivered to support safe and effective care in people with OD and their caregivers?
Which evidence-based strategies should people with OD and their caregivers be taught to reduce meal-related pulmonary risk?

**Table 3:** Summary of Do’s and Don’ts, percentage of agreement, and basis for statement development.

Statement	Round 1	Round 2	Level of Evidence
**DIET PRESCRIPTION: DO’s**			
1. To provide individualized recommendations, bolus trials should follow an ecological criterion that replicates the home mealtime environment as closely as possible, including foods (flavors, colors, smells), consistencies and textures coded using IDDSI terminology, utensils, and environmental supports (e.g., anatomical seating, parent/caregiver presence).	97.8%	100%	V
2. When reporting gold standard instrumental swallowing assessments (FEES, VFSS) and clinical evaluations, it is suggested to specify the volumes, consistencies and textures (using IDDSI terminology) of foods and beverages that allow the person with dysphagia to eat with optimal safety and efficiency.	95.6%	97.7%	V
3. During history taking, it is suggested to investigate the modality used to administer medications, including any need to manipulate solid dosage forms due to OD.	100%	100%	V
4. It is suggested to conduct mealtime observations under the most ecologically valid conditions possible, namely in the person’s usual environment, with the posture, foods, and caregiver(s) that typically characterize their mealtime.	100%	100%	V
5. During mealtime observation, it is suggested to identify environmental and personal factors that act as barriers and facilitators for that specific person.	100%	100%	V
6. To verify the adherence to and the efficacy of the recommendations, one or more follow-up mealtime observations are suggested.	100%	97.7%	V
7. Malnutrition screening should be performed in all patients with OD using validated tools specific to the clinical setting, age group, and population.	80%	97.7%	V
8. It is suggested to diagnose malnutrition through multidimensional assessment criteria.	73.3%	97.7%	III
*9. To identify malnutrition in children with neurological disabilities, the presence of one or more of the following red flags should be investigated: 1. physical signs of undernutrition, such as pressure injuries, skin changes, or impaired peripheral circulation; 2. weight-for-age z-score < −2; 3. tricepts skinfold thickness <10th percentile for age and sex; 4. arm adipose/muscle area <10th percentile; 5. poor growth or failure to thrive.*	57.8%	63.6%	V
*10. It is suggested to perform a comprehensive anthropometric assessment at least every 6 months in children with OD and neurological impairment, together with an annual evaluation of micronutrient status (e.g., vitamin D, iron, calcium, phosphorus).*	66.7%	72.7%	V
11. Nutritional management of OD must ensure both appropriate texture/consistency and adequate energy-protein and fluid intake.	100%	97.7%	III
12. It is suggested to consider the food preferences and cognitive abilities of people with OD to promote adequate intake of the prescribed diet and maximize its acceptability.	100%	97.7%	II
**DIET PRESCRIPTION: DON’Ts**			
13. It is suggested not to perform an instrumental evaluation aimed at diet prescription without a prior clinical assessment that clarifies the indications for the exam and suggests the most appropriate safety conditions.	91.1%	97.7%	V
14. During the first mealtime observation, the clinician should not modify the mealtime conditions or provide instructions, except when necessary for the person’s safety.	100%	97.7%	V
15. It is suggested not to rely solely on patient-, caregiver-, or parent-reported tools, as they cannot independently provide a comprehensive assessment of feeding skills, swallowing function, and mealtime behavior.	97.8%	100%	III
16. It is suggested not to infer energy intake from mealtime observation alone.	95.6%	97.7%	V
17. It is suggested not to base the nutritional and adiposity assessment of a child with neurological impairment solely on weight and height measurements.	80%	88.6%	V
18. It is suggested not to skip periodic nutritional assessments: failure to revise diet prescription according to swallowing function and nutritional status, as well as failure to collect periodic food dietary records, may hinder progress and compromise the safety of the dietary therapy.	100%	93.2%	V
**MEAL PREPARATION: DO’s**			
19. When controlling viscosity, it is important to consider the temperature at which the food or beverage will be consumed, as a decrease in temperature may lead to thickening.	97.8%	100%	III
20. When using starch-containing beverages and starch-based thickeners, it is suggested to consider that viscosity may be significantly reduced by salivary amylase activity.	88.9%	93.2%	V
21. To increase liquid viscosity in adults, the use of xanthan-gum-based thickeners or other amylase-resistant formulations is suggested.	80%	84.1%	I
*22. In case of OD for liquids and constipation, the use of fiber-enriched PHGG gelled water may be considered on an individual basis and in agreement with the treating physician.*	62.2%	72.7%	V
23. To test the texture and consistency of foods and beverages suitable for the person with OD, it is suggested to use the IDDSI framework and terminology.	91.1%	95.5%	V
24. Chewing should be preserved whenever solid foods can be consumed safely, even if only for hedonic purposes.	100%	100%	V
25. Before manipulating solid dosage forms in a person with OD, and always following a medical prescription, it is suggested to evaluate whether an equivalent commercially available medication with marketing authorization can be prescribed in an alternative formulation or administered via a different route.	95.6%	93.2%	V
26. Environmental (physical, social, cultural) factors should be considered to identify intervention areas aimed at improving mealtime management.	97.8%	100%	V
27. When adequate, well-fitted, and accepted by the person, the use of dental prostheses during eating is suggested.	93.3%	95.5%	III
*28. In late pre-term babies, if milk transfer is ineffective, breast compression during sucking may be appropriate, and the use of an ultra-thin silicone nipple shield may be considered.*	57.8%	70.5%	V
29. It is suggested to support the person’s ability to self-feed, including through the use of assistive devices, to promote a sense of competence and maintain self-esteem.	97.8%	100%	V
30. If the person can safely maintain a seated position, with or without assistive devices, it is appropriate to verify and, if necessary, adjust posture before the meal so that they sit on the ischial tuberosities, the trunk is stable, and the feet are fully supported.	97.8%	100%	V
31. In children with OD, an upright or slightly reclined trunk posture is suggested to improve feeding safety and efficiency, although this should be evaluated on a case-by-case basis.	73.3%	86.4%	V
32. A child who has not yet developed functional sitting or who presents with tone abnormalities (spasticity or hypotonia) must be well supported in the highchair or seating system during meals.	93.3%	88.6%	V
**MEAL PREPARATION: DON’Ts**			
33. Do not split tablets into fractions smaller than one-quarter unless explicitly permitted by the manufacturer.	77.8%	79.5%	V
34. Do not feed a person with OD before checking whether head and neck posture interferes with meal safety and efficiency and, if necessary, appropriately adjusting it	100%	97.7%	IV
35. It is suggested not to feed a child with poor trunk control in an unsupported upright position, as this may limit the development of an adequate chewing pattern and increase aspiration risk.	82.2%	84.1%	V
36. It is suggested to limit the use of distractions, such as television, during mealtimes, after verifying that they hinder safety and efficiency.	97.8%	97.7%	V
**MEALTIME EXPERIENCE IN DAILY LIFE: DO’s**			
37. In assessing and managing the implications of swallowing disorders, it is necessary to consider the entire family and social context (family-centered care).	97.8%	97.7%	V
38. It is suggested to frequently share family meals, emphasizing the quality of interaction and allowing the child to observe others consuming healthy foods.	100%	97.7%	V
39. Caregivers should be encouraged to adopt a responsive feeding style that promotes the child’s self-regulation and is associated with healthy eating habits.	97.8%	97.7%	IV
40. The diet of older adults must have qualities that positively influence their quality of life. Foods should be prepared with high-quality ingredients, in ways consistent with the diners’ traditions, and presented in a hygienically safe and aesthetically pleasant setting.	100%	100%	V
41. Foods offered to older adults should stimulate multisensory perception.	100%	100%	V
42. In people with total artificial nutrition, it is suggested to verify whether small amounts of food can be safely consumed for hedonic purposes, while respecting personal preferences and tastes.	100%	100%	V
**MEALTIME EXPERIENCE IN DAILY LIFE: DON’Ts**			
43. In people with OD, the social, symbolic, and psychological aspects of eating as an activity of daily living should not be overlooked.	97.8%	93.2%	V
44. Do not infer the nature and extent of the family and social implications of OD solely from the severity and pathophysiology of the swallowing disorder.	95.6%	97.7%	V
45. It is suggested that caregivers of children with OD should avoid authoritarian, indulgent, or uninvolved feeding styles.	97.8%	90.9%	V
**PATIENT AND CAREGIVER EDUCATION: DO’s**			
46. In quality improvement processes and when optimizing health outcomes, it is appropriate to involve the person with OD, their family, and the community.	100%	97.7%	V
47. Developing interprofessional collaboration can improve outcomes in OD care, as well as the work environment, staff well-being, and organizational functioning.	100%	97.7%	V
48. When educating the person with OD and their informal caregivers, it is suggested to verify learning by asking them to restate information in their own words and provide practical examples, using communication modalities appropriate to the person’s cognitive and linguistic abilities.	97.8%	97.7%	V
49. When educating the person with OD and their informal caregivers, it is suggested to complement written informational materials with video resources, oral explanations, and in-person demonstrations, while avoiding providing excessive information in advance or concentrated in a single session.	100%	100%	V
50. In patients who present episodes of silent aspiration, it is suggested to assess the effectiveness of protective maneuvers (coughing, forced expiration) during meals and investigate potential causes of ineffectiveness.	88.9%	88.6%	V
51. It is suggested to verify and ensure adequate oral hygiene before eating.	95.6%	93.2%	II
52. Assessment of oral health status should preferably be performed by healthcare professionals using validated scales, while considering the clinical condition of the person and documenting the results in the clinical record.	97.8%	100%	V
53. Oral health status must be assessed periodically in all people with known or potential alterations of the oral cavity.	95.6%	100%	V
54. When compatible with the patient’s clinical condition, the use of an electric toothbrush is preferable, as it is more effective at reducing plaque and biofilm compared with a manual toothbrush.	86.7%	77.3%	I
55. Accurate daily oral hygiene at home is suggested to reduce the risk of bacterial colonization and pulmonary complications.	100%	100%	I
56. Oral health and cleanliness of teeth, periodontal tissues, and oral mucosa must be maintained, as these factors facilitate bolus perception and processing during the oral preparatory phase.	100%	100%	V
57. The Free Water Protocol may be used in selected cases, always in conjunction with meticulous oral hygiene and exclusively in settings that ensure strict patient adherence.	66.7%	81.8%	II
**PATIENT AND CAREGIVER EDUCATION: DON’Ts**			
58. To ensure appropriate care delivery, it is suggested to avoid authority imbalances and friction related to “professional boundaries” that may arise from limited understanding of each other’s roles and responsibilities.	86.7%	93.2%	V
59. It is suggested not to use highly specialized scientific terminology without simple explanations, and not to overlook the health literacy level of the person with OD and their caregiver.	97.8%	97.7%	V
60. For adequate oral hygiene, the use of gels or mouthwashes alone is insufficient without the mechanical action of toothbrushing.	93.3%	100%	I
61. Do not use chlorhexidine-based mouthwashes for daily use, except in cases of gingivitis or periodontitis.	73.3%	77.3%	V

Note. The percentage of agreement was calculated based on all respondents, including those who selected the option “unable to express an opinion”. Consensus was defined as agreement ≥75%. Statements that did not reach consensus at Round 2 are reported in Italics. The percentage of agreement reflects the panel’s level of consensus and should not be interpreted as a measure of the certainty of the underlying evidence or as an indicator of the strength with which each statement should be applied in clinical practice. Therefore, high consensus on a statement does not necessarily imply high-quality supporting evidence. For the 33 statements modified after Round 1 (Supplementary Material S4), Round 1 and Round 2 percentages reflect agreement with different versions of the statement and are therefore not directly comparable. Level of evidence of the references supporting the statement was graded according to the Infectious Diseases Society of America–US Public Health Service grading system that was adapted by the European Society for Medical Oncology [47]. The grading system ranged from I (high level of evidence) to V (low level of evidence).

## Data Availability

The original data presented in the study are available in Zenodo (DOI: https://doi.org/10.5281/zenodo.21937543).

## Supplementary Materials

The following supporting information can be downloaded at: https://www.mdpi.com/article/10.3390/nu18193268/s1, Supplementary Material S1: Revisions to the statements after Round 1. Supplementary Material S2: References supporting statements; Supplementary Material S3: Frequency of response options and sensitivity analysis; Supplementary Material S4: Item revision after Round 1; Supplementary Material S5: CREDES checklist.

## Abbreviations

The following abbreviations are used in this manuscript:

ENT	Ear, nose, throat specialist
FEES	Fiberoptic endoscopic evaluation of swallowing
GLIM	Global Leadership Initiative on Malnutrition
ICF	International Classification of Functioning, Disability and Health
IDDSI	International Dysphagia Diet Standardisation Initiative
OD	Oropharyngeal dysphagia
PFD	Pediatric feeding disorders
VFSS	Videofluoroscopic swallowing studies
