# Supplementary material for: Do’s and Don’ts in Mealtime Management for People with Oropharyngeal Dysphagia: A Modified Delphi Consensus Study from the Italian Society of Deglutology
Source: Nutrients. 2026 Oct 4;18(19):3268. doi: 10.3390/nu18193268 (PMC13666940; doi:10.3390/nu18193268)
Supplement: Supplementary file 1 [file nutrients-18-03268-s001.zip › Supplementary material S1. Statements excluded from voting.pdf]

Supplementary Material S1. Statements excluded from voting after review by the panel conveners, with reasons for exclusion (in bold)

DIET PRESCRIPTION - DO'S AND DON'TS

1. A comprehensive swallowing assessment aims to identify the underlying physiological nature of the disorder and to determine the most appropriate interventions. **Not specific to mealtime**
2. Water Swallow Test can be used for dysphagia screening. **Not specific to mealtime**
3. Where possible, reference should be made to a standardized system for classifying consistencies. **Integrated into Statement n.1**
4. At the end of the swallowing assessment, it is suggested to specify the prescribed consistency for foods and beverages using IDDSI terminology. **Integrated into Statement n.2**
5. For FEES to be an ecologically valid examination, it should be performed using the same IDDSI-classified consistencies both in the outpatient setting and at home. **Integrated into Statement n.1.**
6. When choosing the instrumental examination for diagnosing oropharyngeal dysphagia, the advantages of videofluoroscopy should be considered, despite the use of ionizing radiation. **Not specific to mealtime**
7. When reporting videofluoroscopy findings, it is necessary to specify the volume and consistency that allow the patient with dysphagia to be fed safely. **Integrated into Statement n.2**
8. Failing to produce a structured report of the videofluoroscopic examination may compromise nutritional and rehabilitative decision-making for the patient. **Integrated into Statement n.2**
9. Do not omit an assessment of chewing when evaluating the patient with dysphagia. **Not specific to mealtime**
10. During weaning from enteral nutrition to partial or total oral feeding, close clinical and instrumental monitoring (FEES and VFSS) is indicated, as it allows for precise management. **Not specific to mealtime**
11. Do not use BMI as an indicator of adiposity in children with neurological impairment. **Redundant with Statement n.17**
12. Dietary prescription should be carried out by a multidisciplinary team including a specialized dietitian/nutritionist, in order to develop and monitor a personalized nutritional care plan aimed at identifying malnourished patients and/or those at risk of malnutrition. The dietitian supports or guides the development, promotion, and implementation of nutritional management in dysphagia (e.g., dysphagia swallowing screening, specialized diet therapy, meal consistency modification, and use of food for special medical purposes). **Defining the role of individual specialists was outside the scope of the study**

MEAL PREPARATION – DO'S AND DON'TS

13. In people receiving artificial nutrition, capsules containing slow-release or resistant granules may be opened, and the granules suspended in a liquid or, in the case of gastro-resistant granules, in acidic juice. It is suggested to always take the caliber of the feeding tube into consideration. **Not specific to mealtime**
14. The manipulation of solid oral cytostatic dosage forms is absolutely prohibited. **Not specific to mealtime**
15. In children diagnosed with, or at high risk of, cerebral palsy and dysphagia, under 2 years of age, highly reclined positions that could exacerbate swallowing deficits are discouraged. **Redundant with Statement n. 31**
16. Identifying consistency standards for both liquids and solids is necessary to rigorously and effectively guide meal/beverage preparation for the person with dysphagia. **Redundant with Statement n.23.**
17. Determining the correct texture/consistency of foods/beverages for the person with dysphagia at home does not require testing them with a viscometer. **Redundant with Statement n.23**
18. In meals at IDDSI level 5–6, attention must be paid to particle size in relation to the patient's age and abilities. **Redundant with Statement n.23**
19. In meals at IDDSI level 5–6, liquids should never be combined (dual consistencies). **Redundant with Statement n.23**
20. Breastfeeding and human milk are the normative standards for infant feeding and nutrition. The resulting short- and long-term benefits make breastfeeding, or the provision of human milk, a public health imperative. The American Academy of Pediatrics (AAP) recommends exclusive breastfeeding for the first six months of life, the introduction of complementary foods from 6 completed months, and continued breastfeeding until the second year of life and beyond, for as long as mother and child wish. These recommendations are consistent with those of the World Health Organization (WHO). **Not specific to children with dysphagia**
21. In compliance with the International Code of Marketing of Breast-Milk Substitutes, to avoid conflicts of interest and to promote, protect, and support breastfeeding, healthcare professionals must not have relationships with companies producing breast-milk substitutes or artificial teats. **Not specific to mealtime**
22. When a physician prescribes a feeding supplement for a healthy full-term newborn, the use of bottles/artificial teats is not recommended. **Not specific to children with dysphagia**

MEALTIME EXPERIENCE IN DAILY LIFE – DO'S AND DON'TS

23. In the assessment and management of the person with dysphagia, particularly in cases of a chronic disorder, it is suggested to consider the implications of the swallowing disorder on

family and social life, and to adopt specific person-centered and family-centered approaches and strategies. **Redundant with Statements n.37 and n.44**

PATIENT AND CAREGIVER EDUCATION – DO'S AND DON'TS

24. Do not limit interventions to individuals and the team alone when promoting interprofessional collaboration. **Redundant with Statement n.46.**
25. The use of mucolytic agents should be avoided in patients with dysphagia, particularly in the presence of impaired airway defense mechanisms. Where prescription is considered, it should be evaluated with great caution and accompanied by strategies enabling effective and safe clearance of airway secretions. **Not specific to mealtime**
26. In cases of ineffective or absent cough in a patient at risk of aspiration, rehabilitative interventions (e.g., expiratory muscle strength training, improvement of lung volumes and expiratory flow management, biofeedback) or, in any case, compensatory interventions (maneuvers that substitute for coughing or promote airway patency) may be proposed. **Not specific to mealtime**
27. The Free Water Protocol cannot be applied to all patients with oropharyngeal dysphagia. **Redundant with Statement n.57**
28. In the presence of dysphagia to liquids, thorough oral hygiene should not be neglected. **Redundant with Statement n.51**
