# Supplementary material for: Do’s and Don’ts in Mealtime Management for People with Oropharyngeal Dysphagia: A Modified Delphi Consensus Study from the Italian Society of Deglutology
Source: Nutrients. 2026 Oct 4;18(19):3268. doi: 10.3390/nu18193268 (PMC13666940; doi:10.3390/nu18193268)
Supplement: Supplementary file 1 [file nutrients-18-03268-s001.zip › Supplementary material S2. References supporting statements.pdf]

Supplementary Materials S2

Table S2. References provided by the panel experts to support the proposed statements

Statement	References that guided the development of the statement
**DIET PRESCRIPTION: DO's**	
1. To provide individualized recommendations, bolus trials should follow an ecological criterion that replicates the home mealtime environment as closely as possible, including foods (flavors, colors, smells), consistencies and textures coded using IDDSI terminology, utensils, and environmental supports (e.g., anatomical seating, parent/caregiver presence).	Expert opinion only
2. In reporting gold standard instrumental swallowing assessments (FEES, VFSS) and clinical evaluations, it is suggested to specify the volumes, consistencies and textures (using IDDSI terminology) of foods and beverages that allow the person with dysphagia to eat with optimal safety and efficiency.	Steele CM, Alsanei WA, Ayanikalath S, et al. The influence of food texture and liquid consistency modification on swallowing physiology and function: a systematic review. Dysphagia 2015; 30:2.
3. During history taking, it is suggested to investigate the modality used to administer medications, including the need for manipulation of solid dosage forms due to OD.	Carnaby-Mann G, Crary M. Pill swallowing by adults with dysphagia. Arch Otolaryngol Head Neck Surg 2005; 131:970.<br><br>Buhmann C, Bihler M, Emich K, et al. Pill swallowing in Parkinson's disease: A prospective study based on flexible endoscopic evaluation of swallowing. Parkinsonism Relat Disord 2019; 62:51.<br><br>Radhakrishnan C, Sefidani Forough A, Cichero JAY, et al. A Difficult Pill to Swallow: An Investigation of the Factors Associated with Medication Swallowing Difficulties. Patient Prefer Adherence 2021; 15:29.<br><br>Labeit B, Berkovich E, Claus I, Roderigo M, Schwake AL, Izgelov D, et al. Dysphagia for medication in Parkinson's disease. NPJ Parkinsons Dis 2022;8:156.
4. It is suggested to conduct mealtime observation in the most ecological condition possible, meaning in the usual setting, with the posture, foods and any caregiver(s) that typically characterize the person's mealtime.	Hansen T, Fisker Rasmussen SA, Fabricius J, Delgado Grove LM, Simpelaere I. Measuring mealtime performance in older adults with suspected oropharyngeal dysphagia: an updated systematic review of psychometric properties. Disabil Rehabil 2023;1-15.<br><br>Arvedson JC. Assessment of pediatric dysphagia and feeding disorders: clinical and instrumental approaches. Dev Disabil Res Rev 2008;14:118-27.
5. During mealtime observation, it is suggested to identify environmental and personal factors that act as barriers and facilitators to that specific person.	Threats TT. Use of the ICF in dysphagia management. Semin Speech Lang. 2007;28:323-333.

	Nund RL, Scarinci NA, Cartmill B, Ward EC, Kuipers P, Porceddu SV. Application of the International Classification of Functioning, Disability and Health (ICF) to people with dysphagia following non-surgical head and neck cancer management. Dysphagia 2014;29:692-703.<br><br>Speyer R, Cordier R, Farneti D, Nascimento W, Pilz W, Verin E, et al. White Paper by the European Society for Swallowing Disorders: Screening and Non-instrumental Assessment for Dysphagia in Adults. Dysphagia 2022;37(2):333-349.
6. To verify the adherence to and the efficacy of the recommendations, one or more follow-up mealtime observations are suggested.	Expert opinion only
7. Malnutrition screening should be performed in all patients with OD using validated tools specific to the clinical setting, age group, and population.	Kondrup J, Allison SP, Elia M, Vellas B, Plauth M, Educational and Clinical Practice Committee, European Society of Parenteral and Enteral Nutrition (ESPEN). ESPEN guidelines for nutrition screening 2002. Clin Nutr. 2003;22(4):415-421.<br><br>Burgos R, Bretón I, Cereda E, Desport JC, Dziewas R, Genton L, et al. ESPEN guideline clinical nutrition in neurology. Clinical Nutrition. 2018;37(1):354-396.
8. It is suggested to diagnose malnutrition through multidimensional assessment criteria.	Cederholm T, Jensen GL, Correia MITD,et al. GLIM criteria for the diagnosis of malnutrition - A consensus report from the global clinical nutrition community. Clin Nutr. 2019;38(1):1-9.<br><br>Huo Z, Chong F, Yin L, Lu Z, Liu J, Xu H. Accuracy of the GLIM criteria for diagnosing malnutrition: A systematic review and meta-analysis. Clin Nutr. 2022;41(6):1208-1217.<br><br>Alves LF, de Jesus JDS, Britto VNM, de Jesus SA, Santos GS, de Oliveira CC. GLIM criteria to identify malnutrition in patients in hospital settings: A systematic review. JPEN J Parenter Enteral Nutr. 2023;47(6):702-709.
9. To identify malnutrition in children with neurological disabilities, the presence of one or more of the following red flags should be investigated: 1. physical signs of undernutrition such as bedsores, skin changes, impaired peripheral circulation; 2. weight-for-age z-score < -2; 3. triceps skinfold thickness <10th percentile for age and sex; 4. Arm adipose/muscle area <10th percentile; 5. poor growth or failure to thrive.	Stevenson RD, Conaway M, Chumlea WC, et al. Growth and health in children with moderate-to-severe cerebral palsy. Pediatrics 2006;118:1010–8<br><br>Day SM, Strauss DJ, Vachon PJ, et al. Growth patterns in a population of children and adolescents with cerebral palsy. Dev Med Child Neurol 2007;49:167–71.

	Romano C, van Wynckel M, Hulst J, Broekaert I, Bronsky J, Dall'Oglio L, Mis NF, Hojsak I, Orel R, Papadopoulou A, Schaeppi M, Thapar N, Wilschanski M, Sullivan P, Gottrand F. European Society for Paediatric Gastroenterology, Hepatology and Nutrition Guidelines for the Evaluation and Treatment of Gastrointestinal and Nutritional Complications in Children With Neurological Impairment. J Pediatr Gastroenterol Nutr. 2017;65(2):242-264
10. It is suggested to perform a thorough anthropometric assessment at least every 6 months in children with OD and neurological impairment, and an annual evaluation of micronutrient status (e.g., vitamin D, iron, calcium, phosphorus).	Tomoum HY, Badawy NB, Hassan NE, et al. Anthropometry and body composition analysis in children with cerebral palsy. Clin Nutr 2010;29:477–81.<br><br>Hillesund E, Skranes J, Trygg KU, et al. Micronutrient status in children with cerebral palsy. Acta Paediatr 2007;96:1195–8.
11. Nutritional management of OD must ensure both appropriate texture/consistency and adequate energy-protein and fluid intake.	Wright L, Cotter D, Hickson M, Frost G. Comparison of energy and protein intakes of older people consuming a texture modified diet with a normal hospital diet. J Hum Nutr Diet. 2005;18(3):213-219.<br><br>Murray J, Miller M, Doeltgen S, Scholten I. Intake of thickened liquids by hospitalized adults with dysphagia after stroke. Int J Speech Lang Pathol. 2014;16(5):486-494.<br><br>Malagelada, J. R., Bazzoli, F., Boeckxstaens, G., De Looze, D., Fried, M., Kahrilas, P., Lindberg, G., Malfertheiner, P., Salis, G., Sharma, P., Sifrim, D., Vakil, N., & Le Mair, A. (2015). World gastroenterology organisation global guidelines: dysphagia--global guidelines and cascades update September 2014. Journal of clinical gastroenterology, 49(5), 370–378.<br><br>Beck AM, Kjaersgaard A, Hansen T, Poulsen I. Systematic review and evidence based recommendations on texture modified foods and thickened liquids for adults (above 17 years) with oropharyngeal dysphagia - An updated clinical guideline. Clin Nutr. 2018;37(6 Pt A):1980-1991.<br><br>Burgos, R., Bretón, I., Cereda, E., Desport, J. C., Dziewas, R., Genton, L., ... & Bischoff, S. C. (2018). ESPEN guideline clinical nutrition in neurology. Clinical Nutrition, 37(1), 354-396.<br><br>Thibault, R., Abbasoglu, O., Ioannou, E., Meija, L., Ottens-Oussoren, K., Pichard, C., Rothenberg, E., Rubin, D., Siljamäki-Ojansuu, U., Vaillant, M. F., & Bischoff, S.

	C. (2021). ESPEN guideline on hospital nutrition. Clinical nutrition (Edinburgh, Scotland), 40(12), 5684–5709.<br><br>Tomsen N, Bolívar-Prados M, Ortega O, Clavé P. Therapeutic Effect on Swallowing Function and on Hydration Status of a New Liquid Gum-Based Thickener in Independently-Living Older Patients with Oropharyngeal Dysphagia. Nutrients. 2023;15(21):4621.<br><br>Wu XS, Miles A, Braakhuis A. Nutritional Intake and Meal Composition of Patients Consuming Texture Modified Diets and Thickened Fluids: A Systematic Review and Meta-Analysis. Healthcare (Basel). 2020;8(4):579.<br><br>Wu XS, Miles A, Braakhuis AJ. Texture-Modified Diets, Nutritional Status and Mealtime Satisfaction: A Systematic Review. Healthcare (Basel). 2021;9(6):624.
12. It is suggested to consider food preferences and cognitive abilities of people with OD to promote adequate intake of the prescribed diet and maximize acceptability.	Wirth, R., Dziewas, R., Beck, A. M., Clavé, P., Hamdy, S., Heppner, H. J., Langmore, S., Leischker, A. H., Martino, R., Pluschinski, P., Rösler, A., Shaker, R., Warnecke, T., Sieber, C. C., & Volkert, D. (2016). Oropharyngeal dysphagia in older persons - from pathophysiology to adequate intervention: a review and summary of an international expert meeting. Clinical interventions in aging, 11, 189–208.<br><br>Flynn E, Smith CH, Walsh CD, Walshe M. Modifying the consistency of food and fluids for swallowing difficulties in dementia. Cochrane Database Syst Rev. 2018;9:CD011077.<br><br>Ballesteros-Pomar, M. D., Cherubini, A., Keller, H., Lam, P., Rolland, Y., & Simmons, S. F. (2020). Texture-Modified Diet for Improving the Management of Oropharyngeal Dysphagia in Nursing Home Residents: An Expert Review. The journal of nutrition, health & aging, 24(6), 576–581.
**DIET PRESCRIPTION: DON'Ts**	
13. It is suggested not to perform an instrumental evaluation aimed at diet prescription without a prior clinical assessment that clarifies the indications for the exam and suggests the most appropriate safety conditions.	Expert opinion only
14. During the first mealtime observation, the clinician should not modify the mealtime conditions or provide instructions, except when necessary for the person's safety.	Expert opinion only

15. It is suggested not to rely solely on patient-, caregiver-, or parent-reported tools, as they cannot independently provide a comprehensive assessment of feeding skills, swallowing function, and mealtime behavior.	Dewan K, Clarke JO, Kamal AN, Nandwani M, Starmer HM. Patient Reported Outcomes and Objective Swallowing Assessments in a Multidisciplinary Dysphagia Clinic. Laryngoscope. 2021;131(5):1088-1094.<br><br>Pedersen A, Wilson J, McColl E, Carding P, Patterson J. Swallowing outcome measures in head and neck cancer--How do they compare? Oral Oncol. 2016:52:104-8.
16. It is suggested not to infer energy intake from mealtime observation alone.	Expert opinion only
17. It is suggested not to base the nutritional and adiposity assessment of a child with neurological impairment solely on weight and height measurements.	Romano C, van Wynckel M, Hulst J, et al. European Society for Paediatric Gastroenterology, Hepatology and Nutrition Guidelines for the Evaluation and Treatment of Gastrointestinal and Nutritional Complications in Children With Neurological Impairment. J Pediatr Gastroenterol Nutr. 2017 Aug;65(2):242-264
18. It is suggested not to skip periodic nutritional assessments: failure to revise diet prescription in relation to swallowing function and nutritional status, and failure to collect periodic food dietary records, may hinder progress and compromise safety of the dietary therapy.	Burgos, R., Bretón, I., Cereda, E., Desport, J. C., Dziewas, R., Genton, L., ... & Bischoff, S. C. (2018). ESPEN guideline clinical nutrition in neurology. Clinical Nutrition, 37(1), 354-396.<br><br>Ballesteros-Pomar, M. D., Cherubini, A., Keller, H., Lam, P., Rolland, Y., & Simmons, S. F. (2020). Texture-Modified Diet for Improving the Management of Oropharyngeal Dysphagia in Nursing Home Residents: An Expert Review. The journal of nutrition, health & aging, 24(6), 576–581.<br><br>Malagelada, J. R., Bazzoli, F., Boeckxstaens, G., De Looze, D., Fried, M., Kahrilas, P., Lindberg, G., Malfertheiner, P., Salis, G., Sharma, P., Sifrim, D., Vakil, N., & Le Mair, A. (2015). World gastroenterology organisation global guidelines: dysphagia--global guidelines and cascades update September 2014. Journal of clinical gastroenterology, 49(5), 370–378.
**MEAL PREPARATION: DO's**	
19. When controlling viscosity, it is important to consider the temperature at which the food or beverage will be consumed, as a decrease in temperature may lead to thickening.	Hadde EK, Mossel B, Chen J, Prakash S. The safety and efficacy of xanthan gum-based thickeners and their effect in modifying bolus rheology in the therapeutic medical management of dysphagia. Food Hydrocoll Health. 2021;1:100038.

	Vilardell N, Rofes L, Arreola V, Speyer R, Clavé P. A Comparative Study Between Modified Starch and Xanthan Gum Thickeners in Post-Stroke Oropharyngeal Dysphagia. Dysphagia. 2016;31(2):169-179.
20. When using starch-containing beverages and starch-based thickeners, it is suggested to consider that their viscosity may be significantly reduced by salivary enzymes.	Lavoisier A, Boudrag S, Ramaioli M. Effect of α-amylase and pH on the rheological properties of thickened liquids containing starch in in vitro conditions relevant to oral processing and swallowing. J Texture Stud. 2022;53(4):550-557.
21. To increase liquid viscosity in adults, the use of xanthan-gum-based thickeners or other amylase-resistant formulations is suggested.	Leonard RJ, White C, McKenzie S, Belafsky PC. Effects of bolus rheology on aspiration in patients with Dysphagia. J Acad Nutr Diet. 2014;114(4):590-4
22. In case of OD for liquids and constipation, the use of fiber-enriched PHGG gelled water may be considered on an individual basis and in agreement with the treating physician.	Quartarone G. Role of PHGG as a dietary fiber: a review article. Minerva Gastroenterol Dietol. 2013 Dec;59(4):329-40. PMID: 24212352.
23. To test the texture and consistency of foods and beverages suitable for the person with OD, it is suggested to use the IDDSI framework and terminology.	Julie A Y Cichero, Peter Lam , Catriona M Steele , Ben Hanson , Jianshe Chen , Roberto O Dantas , Janice Duivestein , Jun Kayashita , Caroline Lecko , Joseph Murray , Mershen Pillay, Luis Riquelme, Soenke Stanschus Development of International Terminology and Definitions for Texture-Modified Foods and Thickened Fluids Used in Dysphagia Management: The IDDSI Framework. Dysphagia. 2017 Apr;32(2):293-314. doi: 10.1007/s00455-016-9758-y. Epub 2016 Dec 2.<br><br>Hanson B, Steele CM, Lam P, Cichero JAY. Fluid Testing Methods Recommended by IDDSI. Dysphagia. 2019 Oct;34(5):716-717. doi: 10.1007/s00455-018-9957-9. Epub 2018 Oct 31.<br><br>Miles A, Liang V, Sekula J, et al. Texture-modified diets in aged care facilities: Nutrition, swallow safety and mealtime experience. Australas J Ageing 2020; 39:31.<br><br>Sella-Weiss O. What could go wrong? Non-standardized versus standardized food texture classification. Int J Lang Commun Disord. 2022;57(6):1244-1254. doi: 10.1111/1460-6984.12749.
24. Chewing should be preserved in all cases where solid foods can be consumed safely, even for hedonic purposes only.	Kumar A, Almotairy N, Merzo JJ, Wendin K, Rothenberg E, Grigoriadis A, Sandborgh-Englund G, Trulsson M. Chewing and its influence on swallowing, gastrointestinal and nutrition-related factors: a systematic review. Crit Rev Food Sci Nutr. 2023 Nov;63(33):11987-12017. doi: 10.1080/10408398.2022.2098245. Epub 2022. PMID: 35837677.

	Mann T, Heuberger R, Wong H. The association between chewing and swallowing difficulties and nutritional status in older adults. Aust Dent J. 2013;58(2):200-6. doi: 10.1111/adj.12064. PMID: 23713640.
25. Before proceeding with the manipulation of solid dosage forms in a person with OD, and always following a medical prescription, it is suggested to evaluate the possibility of using an equivalent industrially manufactured medication with marketing authorization available in alternative formulations or via different routes of administration.	Italian Ministry of Health. Recommendation for the manipulation of solid oral pharmaceutical forms. Recommendation No. 19. Rome: Ministero della Salute; 2019.<br><br>U.S. Food and Drug Administration. Guidance for industry: tablet scoring - nomenclature, labeling, and data for evaluation. Silver Spring, MD: FDA; 2013. Available from: https://www.fda.gov/media/81626/download
26. Environmental (physical, social, cultural) factors should be considered to identify intervention areas for improving mealtime management.	Kielhofner G, Burke JP. A model of human occupation, part 1. Conceptual framework and content. Am J Occup Ther. 1980;34(9):572-581.<br><br>Strong S, Rigby P, Stewart D, Law M, Letts L, Cooper B. Application of the Person-Environment-Occupation Model: A practical Tool. Can J Occup Ther. 1999;66(3):122-133.
27. When appropriate, well-fitted, and accepted by the person, the use of dental prostheses during eating is suggested.	Sahyoun NR, Krall E. Low dietary quality among older adults with self-perceived ill-fitting dentures. J Am Diet Assoc. 2003;103(11):1494-1499.<br><br>Monaco A, Cattaneo R, Masci C, Spadaro A, Marzo G. Effect of ill-fitting dentures on the swallowing duration in patients using polygraphy. Gerodontology. 2011;29:e637-e644.<br><br>Onodera S, Furuya J, Yamamoto H, Tamada Y, Kondo H. Effects of wearing and removing dentures on oropharyngeal motility during swallowing. J Oral Rehabil. 2016 Nov;43(11):847-854.<br><br>Takagi D, Ohno T, Moriwaki M, Katagiri N, Umeda Y, Tohara H, Nomoto A, Fujishima I. Effect of dentures on pharyngeal swallowing function in patients with dysphagia. Geriatr Gerontol Int. 2021 Oct;21(10):907-912.<br><br>Moynihan P, Varghese R. Impact of Wearing Dentures on Dietary Intake, Nutritional Status, and Eating: A Systematic Review. JDR Clin Trans Res. 2022;7(4):334-351.<br><br>Eggimann AK, Badura L, Zehnder R, Koemeda M, Buser R, Schimmel M. A Comparison of Oral Function in Older In- and Outpatients: An Observational Study. Int J Environ Res Public Health. 2024;21(8):995.

	Sabzevari B, Fatemi A, Soleimani M, Sajedi SM, Babazadehkhoushrodi R. Masticatory performance and oral health related to quality of life before and after orthodontic treatment: a systematic review and meta-analysis. Eur J Transl Myol. 2024;34(1):12101.
28. In late pre-term babies, if milk transfer is ineffective, breast compression during sucking may be appropriate, and the use of an ultra-thin silicone nipple shield may be considered.	Boies EG, Vaucher YE. ABM Clinical Protocol #10: Breastfeeding the Late Preterm (34-36 6/7 Weeks of Gestation) and Early Term Infants (37-38 6/7 Weeks of Gestation), Second Revision 2016. Breastfeed Med. 2016 Dec;11:494-500.
29. It is suggested to support the person's ability to self-feed, including through assistive devices, to promote a sense of competence and maintain self-esteem.	Mandy A, Sims T, Stew G, Onions D. Manual Feeding Device Experiences of People With a Neurodisability. Am J Occup Ther. 2018;72(3):7203345010p1-7203345010p5.
30. If the person can maintain a seated position safely, even with assistive devices, it is appropriate to verify and, if necessary, adjust posture before the meal so that they sit on the ischial tuberosities, the trunk is stable, and the feet are fully supported.	Uesugi Y, Ihara Y, Yuasa K, Takahashi K. Sole-ground contact and sitting leg position influence suprahyoid and sternocleidomastoid muscle activity during swallowing of liquids. Clin Exp Dent Res. 2019;5:505-512.<br><br>Yoshikawa M, Nagakawa K, Tanaka R, et al. Improper sitting posture while eating adversely affects maximum tongue pressure. J Dent Sci. 2021 Jan;16(1):467-473.<br><br>Sakaguchi K, Mehta NR, Maruyama T, Correa LP, Yokoyama A. Effect of sitting posture with and without sole-ground contact on chewing stability and masticatory performance. J Oral Sci. 2023;65(4):251-256. doi: 10.2334/josnusd.23-0172.
31. In children with OD, an upright or slightly reclined trunk posture is suggested to improve feeding safety and efficiency, although it must be evaluated on a case-by-case basis.	Morgan C, Fetters L, Adde L, et al. Early Intervention for Children Aged 0 to 2 Years With or at High Risk of Cerebral Palsy: In-ternational Clinical Practice Guideline Based on Systematic Reviews. JAMA Pediatr. 2021;175(8):846-858.
32. A child who has not yet developed functional sitting or who presents tone abnormalities (spasticity or hypotonia) must be well supported in the highchair or seating system during meals.	Jackson BN, Helier T, Exley K, Hampton Edwards B, Johnston S, Keesing M, et al. New Zealand Speech-Language Therapy Clinical Practice Guideline on Children's Feeding and Swallowing Difficulties (Paediatric Dysphagia). Wellington: New Zealand Speech-Language Therapists' Association; 2019.
**MEAL PREPARATION: DON'Ts**	
33. Do not split tablets into fractions smaller than one-quarter unless explicitly permitted by the manufacturer.	U.S. Food and Drug Administration. Guidance for industry: tablet scoring - nomenclature, labeling, and data for evaluation. Silver Spring, MD: FDA; 2013. Available from: https://www.fda.gov/media/81626/download
34. Do not feed a person with OD before checking and, if necessary, appropriately adjusting head and neck posture when these interfere with meal safety and efficiency.	Yamada R, Ogawa T, Koyano K. The effect of head posture on direction and stability of mandibular closing movement. J Oral Rehabil. 1999;26(6):511-520.

	Nakamura T, Kita Y, Fujimoto J, Ayuzawa K, Ozawa H. Hyoid bone movement during swallowing and mechanism of pharyngeal residue in patients with profound intellectual and multiple disabilities. Int J Pediatr Otorhinolaryngol. 2021;149:110849.<br><br>Suzuki T, Hino H, Magara J, Tsujimura T, Ito K. Effects of Head and Neck Alignment and Pharyngeal Anatomy on Epiglottic Inversion During Swallowing in Dysphagic Patients. Dysphagia. 2023;38(6):1519-1527.
35. It is suggested not to feed a child with poor trunk control in an unsupported upright position, as this may limit the development of an adequate chewing pattern and increase aspiration risk.	Jackson BN, Helier T, Exley K, Hampton Edwards B, Johnston S, Keesing M, et al. New Zealand Speech-Language Therapy Clinical Practice Guideline on Children's Feeding and Swallowing Difficulties (Paediatric Dysphagia). Wellington: New Zealand Speech-Language Therapists' Association; 2019.
36. It is suggested to limit the use of distractions, such as television, during mealtimes, after verifying that they hinder safety and efficiency.	Scaglioni S, De Cosmi V, Ciappolino V, Parazzini F, Brambilla P, Agostoni C. Factors influencing children's eating behaviours. Nutrients. 2018;10(6):706.<br><br>Zimmerman S, Sloane PD, Cohen LW, et al. Nursing assistants' opinions on creating the perfect dining experience for residents with dementia. Gerontologist. 2019;59(Suppl 1):S40-S48.<br><br>Fisberg M, Gioia N, Maximino P. Transgenerational transmission of eating habits. J Pediatr (Rio J). 2024;100(Suppl 1):S82-S87.
**MEALTIME EXPERIENCE IN DAILY LIFE: DO's**	
37. In assessing and managing the implications of swallowing disorders, it is necessary to include the entire family and social context (family-centered care).	Bayne DF, Shune SE. A Biopsychosocial Model of Mealtime Management in Persons with Dementia, an Asset-Based Approach to Patient-Centered Care. Geriatrics (Basel). 2022;7(5):112.
38. It is suggested to frequently share family meals, emphasizing the quality of interaction and allowing the child to observe others consuming healthy foods.	Fisberg M, Gioia N, Maximino P. Transgenerational transmission of eating habits. J Pediatr (Rio J). 2024 Mar-Apr;100 Suppl 1(Suppl 1):S82-S87. doi: 10.1016/j.jped.2023.11.007. Epub 2023 Dec 22. PMID: 38142715; PMCID: PMC10960189.<br><br>Haines J, Haycraft E, Lytle L, Nicklaus S, Kok FJ, Merdji M, et al. Nurturing children's healthy eating: position statement. Appetite. 2019;137:124-33.<br><br>Scaglioni S, De Cosmi V, Ciappolino V, Parazzini F, Brambilla P, Agostoni C. Factors Influencing Children's Eating Behaviours. Nutrients. 2018 May 31;10(6):706. doi: 10.3390/nu10060706. PMID: 29857549; PMCID: PMC6024598.
39. Caregivers should be encouraged to adopt a responsive feeding style that promotes the child's self-regulation and is associated with healthy eating habits.	Fisberg M, Gioia N, Maximino P. Transgenerational transmission of eating habits. J Pediatr (Rio J). 2024 Mar-Apr;100 Suppl 1(Suppl 1):S82-S87. doi:

	10.1016/j.jped.2023.11.007. Epub 2023 Dec 22. PMID: 38142715; PMCID: PMC10960189.<br><br>Finistrella V, Gianni N, Fintini D, Menghini D, Amendola S, Donini LM, Manco M. Neophobia, sensory experience and child's schemata contribute to food choices. Eat Weight Disord. 2024 Apr 8;29(1):25. doi: 10.1007/s40519-024-01657-5. PMID: 38587606; PMCID: PMC11001701.<br><br>Scaglioni S, De Cosmi V, Ciappolino V, Parazzini F, Brambilla P, Agostoni C. Factors Influencing Children's Eating Behaviours. Nutrients. 2018 May 31;10(6):706. doi: 10.3390/nu10060706. PMID: 29857549; PMCID: PMC6024598.<br><br>Haines J, Haycraft E, Lytle L, Nicklaus S, Kok FJ, Merdji M, et al. Nurturing children ' s healthy eating: position statement. Appetite. 2019;137:124 - 33.
40. The diet of older adults must have qualitative characteristics that positively influence their quality of life. Foods should be prepared with high-quality ingredients, in ways consistent with the diners' traditions, and presented in a hygienically safe and aesthetically pleasant setting.	Leitão C, Mignano A, Estrela M, Fardilha M, Figueiras A, Roque F, Herdeiro MT. The Effect of Nutrition on Aging-A Systematic Review Focusing on Aging-Related Biomarkers. Nutrients. 2022 Jan 27;14(3):554. doi: 10.3390/nu14030554. PMID: 35276919; PMCID: PMC8838212.
41. Foods offered to older adults should be able to stimulate multisensory perception.	Delwiche J. The impact of perceptual interactions on perceived flavor. Food Quality and Preference. 2004;15:137-146.<br><br>Chen L, Hemsley B, Debono D. The impact of food-shaping techniques on nutrition, mealtime experiences, and quality of life for older adults in aged care settings: a systematic review. Curr Nutr Rep. 2024;13(1):1-18.
42. In people with total artificial nutrition, it is suggested to verify whether small amounts of food can be safely consumed for hedonic purposes, respecting personal preferences and tastes.	Soar N, Birns J, Sommerville P, Lang A, Archer S. Approaches to eating and drinking with acknowledged risk: a systematic review. Dysphagia. 2021;36(1):54-66. doi: 10.1007/s00455-020-10107-0.
**MEALTIME EXPERIENCE IN DAILY LIFE: DON'Ts**	
43. In people with OD, social, symbolic, and psychological aspects of eating as an activity of daily living should not be overlooked.	Cipriano-Crespo C, Rodríguez-Hernández M, Cantero-Garlito P, Mariano-Juárez L. Eating Experiences of People with Disabilities: A Qualitative Study in Spain. Healthcare (Basel). 2020;8(4):512.<br><br>Bartlett RS, Walters AS, Wayment HA. Psychosocial experiences associated with dysphagia and relevant clinical implications among adults with Parkinson disease. Dysphagia. 2025;40(1):231-247.

	Namasivayam-MacDonald AM, Shune SE. The burden of dysphagia on family caregivers of the elderly: A systematic review. Geriatrics (Basel). 2018;3(2):30.
44. Do not infer the nature and extent of the family and social implications of OD solely from the severity and pathophysiology of the swallowing disorder.	Ninfa A, Jansen F, Delle Fave A, et al. The Change in Social Eating over Time in People with Head and Neck Cancer Treated with Primary (Chemo)Radiotherapy: The Role of Swallowing, Oral Function, and Nutritional Status. Cancers (Basel). 2023;15(5):1603.<br><br>Vermaire JA, Raaijmakers CPJ, Verdonck-de Leeuw IM, et al. Mastication, swallowing, and salivary flow in patients with head and neck cancer: objective tests versus patient-reported outcomes. Support Care Cancer. 2021;29(12):7793-7803.
45. It is suggested that caregivers of children with OD should avoid authoritarian, indulgent, or uninvolved feeding styles.	Haines J, Haycraft E, Lytle L, Nicklaus S, Kok FJ, Merdji M, et al. Nurturing children's healthy eating: position statement. Appetite. 2019;137:124 - 33.
**PATIENT AND CAREGIVER EDUCATION: DO's**	
46. In quality improvement processes and in optimizing health outcomes, it is appropriate to involve the person with OD, their family, and the community.	Jadotte YT, Noel K. Definitions and core competencies for interprofessional education in telehealth practice. Clin Integrat Care. 2021;6:100054.
47. Developing interprofessional collaboration can improve outcomes in OD care, as well as in the work environment, staff well-being, and organizational functioning.	Zwarenstein M, Goldman J, Reeves S. Interprofessional collaboration: effects of practice-based interventions on professional practice and healthcare outcomes. Cochrane Database Syst Rev. 2009;3:CD000072.<br><br>Reeves S, Pelone F, Harrison R, Goldman J, Zwarenstein M. Interprofessional collaboration to improve professional practice and healthcare outcomes. Cochrane Database Syst Rev. 2017;6:CD000072<br><br>Guck TP, Potthoff MR, Walters RW, Doll J, Greene MA, DeFreece T. Improved outcomes associated with interprofessional collaborative practice. Ann Fam Med. 2019;17(Suppl 1):S82.<br><br>Wei H, Horns P, Sears SF, et al. A systematic meta-review of systematic reviews about interprofessional collaboration: facilitators, barriers, and outcomes. J Interprof Care. 2022;36(5):735-749.

48. When educating the person with OD and their informal caregivers, it is suggested to verify learning by asking them to restate information in their own words and provide practical examples, using communication modalities appropriate to the person's cognitive and linguistic abilities.	Govender, R., Smith, C. H., Taylor, S. A., Barratt, H., & Gardner, B. (2017). Swallowing interventions for the treatment of dysphagia after head and neck cancer: A systematic review of behavioural strategies used to promote patient adherence to swallowing exercises. BMC Cancer, 17(1), 43.<br><br>Ha Dinh TT, Bonner A, Clark R, Ramsbotham J, Hines S. The effectiveness of the teach-back method on adherence and self-management in health education for people with chronic disease: a systematic review. JBI Database System Rev Implement Rep. 2016 Jan;14(1):210-47.
49. When educating the person with OD and their informal caregivers, it is suggested to complement written informational materials with video resources, oral explanations, and in-person demonstrations, and to avoid providing excessive information in advance or concentrated in a single session.	Govender, R., Taylor, S. A., Smith, C. H., & Gardner, B. (2019). Helping Patients With Head and Neck Cancer Understand Dysphagia: Exploring the Use of Video-Animation. American Journal of Speech-Language Pathology, 28(2), 697–705.<br><br>Jennebach J, Ahlers O, Simonsohn A, Adler M, Özkaya J, Raupach T, Fischer MR. Digital patient-centred learning in medical education: A national learning platform with virtual patients as part of the DigiPaL project. GMS J Med Educ. 2022;39(4):Doc47.
50. In patients who present episodes of silent aspiration, it is suggested to assess the effectiveness of protective maneuvers (coughing, forced expiration) during meals and investigate potential causes of ineffectiveness.	Dallal-York J, Troche MS. Hypotussic cough in persons with dysphagia: biobehavioral interventions and pathways to clinical implementation. Front Rehabil Sci. 2024 Jun 12;5:1394110. doi: 10.3389/fresc.2024.1394110. PMID: 38933659; PMCID: PMC11199739.<br><br>Lowell ER, Borders JC, Sevitz JS, Dakin AE, Brates D, Troche MS. A primer on hypotussic cough: mechanisms and assessment. Curr Otorhinolaryngol Rep. (2023) 11 (2):182–91. doi: 10.1007/s40136-023-00446-5<br><br>Palmer PM, Padilla AH. Risk of an adverse event in individuals who aspirate: a review of current literature on host defenses and individual differences. Am J Speech Lang Pathol. (2022) 31(1):148–62. doi: 10.1044/2021_AJSLP-20-00375<br><br>Jamróz B, Sobol M, Chmielewska-Walczak J, Milewska M, Niemczyk K. The risk factors for silent aspiration: A retrospective case series and literature review. Int J Lang Commun Disord. 2024 Jul-Aug;59(4):1538-1552.
51. It is suggested to verify and ensure adequate oral hygiene before food intake.	Quagliarello V, Ginter S, Han L, Van Ness P, Allore H, Tinetti M. Modifiable risk factors for nursing home-acquired pneumonia. Clin Infect Dis. 2005 Jan 1;40(1):1-6.

	Bassim CW, Gibson G, Ward T, Paphides BM, Denucci DJ. Modification of the risk of mortality from pneumonia with oral hygiene care. J Am Geriatr Soc. 2008 Sep;56(9):1601-7<br><br>Son M, Jo S, Lee JS, Lee DH. Association between oral health and incidence of pneumonia: a population-based cohort study from Korea. Sci Rep. 2020 Jun 12;10(1):9576.<br><br>Dziewas R, Michou E, Trapl-Grundschober M, Lal A, Arsava EM, Bath PM, et al. European Stroke Organisation and European Society for Swallowing Disorders guideline for the diagnosis and treatment of post-stroke dysphagia. Eur Stroke J. 2021;6(3):LXXXIX-CXV.<br><br>Tehan PE, Browne K, Matterson G, Cheng AC, Dawson S, Graves N, Johnson D, Kiernan M, Madhuvu A, Marshall C, McDonagh J, Northcote M, O'Connor J, Orr L, Rawson H, Russo P, Sim J, Stewardson AJ, Wallace J, White N, Wilson R, Mitchell BG. Oral care practices and hospital-acquired pneumonia prevention: A national survey of Australian nurses. Infect Dis Health. 2024 Nov;29(4):212-217.
52. Assessment of oral health status should preferably be performed by healthcare professionals using validated scales, considering the clinical condition of the person and documenting results in the clinical record.	Thapa R, Chimoriya R, Arora A. The development and psychometric properties of oral health assessment instruments used by non-dental professionals for nursing home residents: a systematic review. BMC Geriatr. 2021 Jan 9;21(1):35.<br><br>Chou KR, Huang MS, Chiu WC, Chen YH, Chen YY, Xiao Q, Yang SC. A comprehensive assessment of oral health, swallowing difficulty, and nutritional status in older nursing home residents. Sci Rep. 2023 Nov 14;13(1):19914.
53. Oral health status must be assessed periodically in all people with known or potential alterations of the oral cavity.	Collins T, Plowright C, Gibson V, Stayt L, Clarke S, Caisley J, Watkins CH, Hodges E, Leaver G, Leyland S, McCready P, Millin S, Platten J, Scallon M, Tipene P, Wilcox G. British Association of Critical Care Nurses: Evidence-based consensus paper for oral care within adult critical care units. Nurs Crit Care. 2021 Jul;26(4):224-233.<br><br>Oda K, Majeed S, Parsons J, Boyd M, Smith M. Putting the Mouth Into the Head-to-Toe Assessment: Nursing Oral Health Assessment Training With an Oral Health Therapist. J Nurs Educ. 2023 Jul;62(7):399-402.

54. When compatible with the patient's clinical condition, the use of an electric toothbrush is preferable, as it has greater efficacy in reducing plaque and biofilm compared with a manual toothbrush.	Munirah Yaaco, Helen V Worthington, Scott A Deacon, Chris Deery, A Damien Walmsley, Peter G Robinson, Anne-Marie Glenny. Powered versus manual toothbrushing for oral health. Cochrane Database Syst Rev. 2014 Jun 17;2014(6):CD002281.
55. Performing accurate daily oral hygiene at home is suggested to reduce the risk of bacterial colonization and pulmonary complications.	Yoneyama T, Yoshida M, Ohrui T, Mukaiyama H, Okamoto H, Hoshiba K, Ihara S, Yanagisawa S, Ariumi S, Morita T, Mizuno Y, Ohsawa T, Akagawa Y, Hashimoto K, Sasaki H; Oral Care Working Group. Oral care reduces pneumonia in older patients in nursing homes. J Am Geriatr Soc. 2002 Mar;50(3):430-3.
56. Oral health and cleanliness of teeth, periodontal tissues, and oral mucosa must be maintained, as they facilitate bolus perception and processing during the oral preparatory phase.	Ahn-Jarvis JH, Piancino MG. Chapter 14: Impact of Oral Health on Diet/Nutrition. Monogr Oral Sci. 2020;28:134-147.<br><br>Piancino MG, Isola G, Cannavale R, Cutroneo G, Vermiglio G, Bracco P, Anastasi GP. From periodontal mechanoreceptors to chewing motor control: A systematic review. Arch Oral Biol. 2017;78:109-121.
57. The Free Water Protocol may be used in selected cases, always in conjunction with meticulous oral hygiene and exclusively in settings that ensure strict patient adherence.	Gillman A, Winkler R, Taylor NF. Implementing the Free Water Protocol does not Result in Aspiration Pneumonia in Carefully Selected Patients with Dysphagia: A Systematic Review. Dysphagia. 2017 Jun;32(3):345-361.<br><br>Sedar J, Penn C. Implementing oral care to reduce aspiration pneumonia amongst patients with dysphagia in a South African setting. S Afr J Commun Disorder. 2016;63(1):102.
**PATIENT AND CAREGIVER EDUCATION: DON'Ts**	
58. To ensure appropriate care delivery, it is suggested to avoid authority imbalances and friction between "professional boundaries" arising from limited understanding of each other's roles and responsibilities.	Wei H, Horns P, Sears SF, et al. A systematic meta-review of systematic reviews about interprofessional collaboration: facilitators, barriers, and outcomes. J Interprof Care. 2022;36(5):735-749.
59. It is suggested not to use highly specialized scientific terminology without simple explanations, and not to overlook the health literacy level of the person with OD and their caregiver.	Berkman ND, Sheridan SL, Donahue KE, et al. Health literacy interventions and outcomes: an updated systematic review. Ann Intern Med. 2011;155(2):97-107.<br><br>Joseph G, Lee R, Pasick RJ, Guerra C, Schillinger D, Rubin S. Effective communication in the era of precision medicine: A pilot intervention with low health literacy patients to improve genetic counseling communication. Eur J Med Genet. 2019;62(5):357-367.<br><br>Ghaddar Z, Matar N, Noujaim J, Diep AN, Tohmé A, Pétré B. Consensus on the objectives of an educational intervention for patients with oropharyngeal

	dysphagia and their informal caregivers: a Delphi study. Patient Prefer Adherence. 2022;16:1511-1524.
60. For adequate oral hygiene, the use of gels or mouthwashes alone is insufficient without the mechanical action of toothbrushing.	Silva PUJ, Paranhos LR, Meneses-Santos D, et al. Combination of toothbrushing and chlorhexidine compared with exclusive use of chlorhexidine to reduce the risk of ventilator-associated pneumonia: a systematic review with meta-analysis. Clinics (Sao Paulo). 2021;76:e2659.
61. Do not use chlorhexidine-based mouthwashes for daily use, except in cases of gingivitis or periodontitis.	Brookes ZLS, Bescos R, Belfield LA, Ali K, Roberts A. Current uses of chlorhexidine for management of oral disease: a narrative review. J Dent. 2020;103:103497.
