# Supplementary material for: Do’s and Don’ts in Mealtime Management for People with Oropharyngeal Dysphagia: A Modified Delphi Consensus Study from the Italian Society of Deglutology
Source: Nutrients. 2026 Oct 4;18(19):3268. doi: 10.3390/nu18193268 (PMC13666940; doi:10.3390/nu18193268)
Supplement: Supplementary file 1 [file nutrients-18-03268-s001.zip › Supplementary Material S3. Frequency of response options and sensitivity analysis (clear).pdf]

Supplementary Material S3

Table S3. Frequency of response options at Round 1 and Round 2 and sensitivity analysis excluding "unable to express an opinion" responses from the denominator

Statement	Round 1					Round 2				
	% Agree	% Agree with reservations	% Disagree	% Unable to express an opinion	Sensitivity analysis % agreement	% Agree	% Agree with reservations	% Disagree	% Unable to express an opinion	Sensitivity analysis % agreement
**DIET PRESCRIPTION: DO's**										
1. To provide individualized recommendations, bolus trials should follow an ecological criterion that replicates the home mealtime environment as closely as possible, including foods (flavors, colors, smells), consistencies and textures coded using IDDSI terminology, utensils, and environmental supports (e.g., anatomical seating, parent/caregiver presence).	93.3%	4.4%	0%	2.2%	100%	100%	0%	0%	0%	100%
2. When reporting gold standard instrumental swallowing assessments (FEES, VFSS) and clinical evaluations, it is suggested to specify the volumes, consistencies and textures (using IDDSI terminology) of foods and beverages that allow the person with dysphagia to eat with optimal safety and efficiency	93.3%	2.2%	0%	4.4%	100%	97.7%	0%	0%	2.3%	100%
3. During history taking, it is suggested to investigate the modality used to administer medications, including any need to manipulate solid dosage forms due to OD.	100%	0%	0%	0%	100%	100%	0%	0%	0%	100%
4. It is suggested to conduct mealtime observations under the most ecologically valid conditions possible, namely in the person's usual environment, with the posture, foods, and caregiver(s) that typically characterize their mealtime.	95.6%	4.4%	0%	0%	100%	100%	0%	0%	0%	100%

5. During mealtime observation, it is suggested to identify environmental and personal factors that act as barriers and facilitators for that specific person.	93.3%	6.7%	0%	0%	100%	100%	0%	0%	0%	100%
6. To verify the adherence to and the efficacy of the recommendations, one or more follow-up mealtime observations are suggested.	97.8%	2.2%	0%	0%	100%	97.7%	0%	2.3%	0%	97.7%
7. Malnutrition screening should be performed in all patients with OD using validated tools specific to the clinical setting, age group, and population.	68.9%	11.1%	0%	20%	100%	97.7%	0%	2.3%	0%	97.7%
8. It is suggested to diagnose malnutrition through multidimensional assessment criteria.	62.2%	11.1%	0%	26.7%	100%	97.7%	0%	0%	2.3%	100%
9. To identify malnutrition in children with neurological disabilities, the presence of one or more of the following red flags should be investigated: 1. physical signs of undernutrition, such as pressure injuries, skin changes, or impaired peripheral circulation; 2. weight-for-age z-score < -2; 3. triceps skinfold thickness <10th percentile for age and sex; 4. arm adipose/muscle area <10th percentile; 5. poor growth or failure to thrive.	46.7%	11.1%	2.2%	40%	96.3%	59%	4.5%	0%	36.4%	100%
10. It is suggested to perform a comprehensive anthropometric assessment at least every 6 months in children with OD and neurological impairment, together with an annual evaluation of micronutrient status (e.g., vitamin D, iron, calcium, phosphorus).	60%	6.7%	0%	33.3%	100%	70.5%	2.3%	0%	27.3%	100%
11. Nutritional management of OD must ensure both appropriate texture/consistency and adequate energy-protein and fluid intake.	97.8%	2.2%	0%	0%	100%	97.7%	0%	0%	2.3%	100%
12. It is suggested to consider the food preferences and cognitive abilities of people with OD to promote adequate	86.7%	13.3%	0%	0%	100%	95.5%	2.3%	0%	2.3%	100%

intake of the prescribed diet and maximize its acceptability.										
**DIET PRESCRIPTION: DON'Ts**										
13. It is suggested not to perform an instrumental evaluation aimed at diet prescription without a prior clinical assessment that clarifies the indications for the exam and suggests the most appropriate safety conditions.	88.9%	2.2%	4.4%	4.4%	95.3%	97.7%	0%	0%	2.3%	100%
14. During the first mealtime observation, the clinician should not modify the mealtime conditions or provide instructions, except when necessary for the person's safety.	93.3%	6.7%	0%	0%	100%	97.7%	0%	0%	2.3%	100%
15. It is suggested not to rely solely on patient-, caregiver-, or parent-reported tools, as they cannot independently provide a comprehensive assessment of feeding skills, swallowing function, and mealtime behavior.	88.9%	8.9%	0%	2.2%	100%	97.7%	2.3%	0%	0%	100%
16. It is suggested not to infer energy intake from mealtime observation alone.	93.3%	2.2%	2.2%	2.2%	97.7%	97.7%	0%	0%	2.3%	100%
17. It is suggested not to base the nutritional and adiposity assessment of a child with neurological impairment solely on weight and height measurements.	64.4%	15.6%	2.2%	17.8%	97.3%	84%	4.5%	0%	11.4%	100%
18. It is suggested not to skip periodic nutritional assessments: failure to revise diet prescription according to swallowing function and nutritional status, as well as failure to collect periodic food dietary records, may hinder progress and compromise the safety of the dietary therapy.	100%	0%	0%	0%	100%	93.2%	0%	0%	6.8%	100%
**MEAL PREPARATION: DO's**										
19. When controlling viscosity, it is important to consider the temperature at which the food or beverage will be	93.3%	4.4%	0%	2.2%	100%	100%	0%	0%	0%	100%

consumed, as a decrease in temperature may lead to thickening.										
20. When using starch-containing beverages and starch-based thickeners, it is suggested to consider that viscosity may be significantly reduced by salivary amylase activity.	82.2%	6.7%	0%	11.1%	100%	93.2%	0%	0%	6.8%	100%
21. To increase liquid viscosity in adults, the use of xanthan-gum-based thickeners or other amylase-resistant formulations is suggested.	66.7%	13.3%	2.2%	17.8%	97.3%	81.8%	2.3%	2.3%	13.7%	97.4%
22. In case of OD for liquids and constipation, the use of fiber-enriched PHGG gelled water may be considered on an individual basis and in agreement with the treating physician.	57.8%	4.4%	4.4%	33.3%	93.3%	72.7%	0%	4.5%	22.7%	94.1%
23. To test the texture and consistency of foods and beverages suitable for the person with OD, it is suggested to use the IDDSI framework and terminology.	88.9%	2.2%	0%	8.9%	100%	95.5%	0%	0%	4.5%	100%
24. Chewing should be preserved whenever solid foods can be consumed safely, even if only for hedonic purposes.	95.6%	4.4%	0%	0%	100%	100%	0%	0%	0%	100%
25. Before manipulating solid dosage forms in a person with OD, and always following a medical prescription, it is suggested to evaluate whether an equivalent commercially available medication with marketing authorization can be prescribed in an alternative formulation or administered via a different route.	93.3%	4.4%	2.2%	0%	97.8%	93.2%	0%	0%	6.8%	93.2%
26. Environmental (physical, social, cultural) factors should be considered to identify intervention areas aimed at improving mealtime management.	57.8%	15.6%	0%	26.7%	100%	100%	0%	0%	0%	100%
27. When adequate, well-fitted, and accepted by the person, the use of dental prostheses during eating is suggested.	86.7%	6.7%	0%	6.7%	100%	93.2%	2.3%	2.3%	2.3%	97.7%

28. In late pre-term babies, if milk transfer is ineffective, breast compression during sucking may be appropriate, and the use of an ultra-thin silicone nipple shield may be considered.	55.6%	2.2%	0%	42.2%	100%	70.5%	0%	0%	29.5%	100%
29. It is suggested to support the person's ability to self-feed, including through the use of assistive devices, to promote a sense of competence and maintain self-esteem.	95.6%	2.2%	0%	2.2%	100%	100%	0%	0%	0%	100%
30. If the person can safely maintain a seated position, with or without assistive devices, it is appropriate to verify and, if necessary, adjust posture before the meal so that they sit on the ischial tuberosities, the trunk is stable, and the feet are fully supported.	88.9%	8.9%	0%	2.2%	100%	97.7%	2.3%	0%	0%	100%
31. In children with OD, an upright or slightly reclined trunk posture is suggested to improve feeding safety and efficiency, although this should be evaluated on a case-by-case basis.	57.8%	15.6%	0%	26.7%	73.3%	81.8%	4.5%	2.3%	11.4%	97.4%
32. A child who has not yet developed functional sitting or who presents with tone abnormalities (spasticity or hypotonia) must be well supported in the highchair or seating system during meals.	93.3%	0%	0%	6.7%	100%	88.6%	0%	0%	11.4%	100%
**MEAL PREPARATION: DON'Ts**										
33. Do not split tablets into fractions smaller than one-quarter unless explicitly permitted by the manufacturer.	75.6%	2.2%	0%	22.2%	100%	79.5%	0%	2.3%	18.2%	97.2%
34. Do not feed a person with OD before checking whether head and neck posture interferes with meal safety and efficiency and, if necessary, appropriately adjusting it.	88.9%	11.1%	0%	0%	100%	93.2%	4.5%	2.3%	0%	97.7%
35. It is suggested not to feed a child with poor trunk control in an unsupported upright position, as this may limit the	80%	2.2%	0%	17.8%	100%	84.1%	0%	0%	15.9%	100%

development of an adequate chewing pattern and increase aspiration risk.										
36. It is suggested to limit the use of distractions, such as television, during mealtimes, after verifying that they hinder safety and efficiency.	86.7%	8.9%	0%	4.4%	100%	97.7%	0%	0%	2.3%	100%
**MEALTIME EXPERIENCE IN DAILY LIFE: DO's**										
37. In assessing and managing the implications of swallowing disorders, it is necessary to consider the entire family and social context (family-centered care).	95.6%	4.4%	0%	0%	100%	97.7%	0%	0%	2.3%	100%
38. It is suggested to frequently share family meals, emphasizing the quality of interaction and allowing the child to observe others consuming healthy foods.	97.8%	2.2%	0%	0%	100%	97.7%	0%	0%	2.3%	100%
39. Caregivers should be encouraged to adopt a responsive feeding style that promotes the child's self-regulation and is associated with healthy eating habits.	95.6%	2.2%	0%	2.2%	100%	97.7%	0%	0%	2.3%	100%
40. The diet of older adults must have qualities that positively influence their quality of life. Foods should be prepared with high-quality ingredients, in ways consistent with the diners' traditions, and presented in a hygienically safe and aesthetically pleasant setting.	95.6%	4.4%	0%	0%	100%	97.7%	2.3%	0%	0%	100%
41. Foods offered to older adults should stimulate multisensory perception.	95.6%	4.4%	0%	0%	100%	100%	0%	0%	0%	100%
42. In people with total artificial nutrition, it is suggested to verify whether small amounts of food can be safely consumed for hedonic purposes, while respecting personal preferences and tastes.	93.3%	6.7%	0%	0%	100%	97.7%	2.3%	0%	0%	100%
**MEALTIME EXPERIENCE IN DAILY LIFE: DON'Ts**										
43. In people with OD, the social, symbolic, and psychological aspects of	93.3%	4.4%	2.2%	0%	97.8%	93.2%	0%	2.3%	4.5	97.6%

eating as an activity of daily living should not be overlooked.										
44. Do not infer the nature and extent of the family and social implications of OD solely from the severity and pathophysiology of the swallowing disorder.	91.1%	4.4%	0%	4.4%	100%	97.7%	0%	0%	2.3%	100%
45. It is suggested that caregivers of children with OD should avoid authoritarian, indulgent, or uninvolved feeding styles.	86.7%	11.1%	0%	2.2%	100%	86.4%	4.5%	0%	9.1%	100%
**PATIENT AND CAREGIVER EDUCATION: DO's**										
46. In quality improvement processes and when optimizing health outcomes, it is appropriate to involve the person with OD, their family, and the community.	97.8%	2.2%	0%	0%	100%	97.7%	0%	0%	2.3%	100%
47. Developing interprofessional collaboration can improve outcomes in OD care, as well as the work environment, staff well-being, and organizational functioning.	97.8%	2.2%	0%	0%	100%	97.7%	0%	0%	2.3%	100%
48. When educating the person with OD and their informal caregivers, it is suggested to verify learning by asking them to restate information in their own words and provide practical examples, using communication modalities appropriate to the person's cognitive and linguistic abilities.	88.9%	8.9%	2.2%	0%	97.8%	95.5%	2.3%	0%	2.3%	100%
49. When educating the person with OD and their informal caregivers, it is suggested to complement written informational materials with video resources, oral explanations, and in-person demonstrations, while avoiding providing excessive information in advance or concentrated in a single session.	95.6%	4.4%	0%	0%	100%	100%	0%	0%	0%	100%

50. In patients who present episodes of silent aspiration, it is suggested to assess the effectiveness of protective maneuvers (coughing, forced expiration) during meals and investigate potential causes of ineffectiveness.	84.4%	4.4%	4.4%	6.7%	95.2%	86.4%	2.3%	2.3%	9.1%	97.5%
51. It is suggested to verify and ensure adequate oral hygiene before eating	91.1%	6.7%	0%	2.2%	100%	90.1%	2.3%	4.5%	2.3%	95.3%
52. Assessment of oral health status should preferably be performed by healthcare professionals using validated scales, while considering the clinical condition of the person and documenting the results in the clinical record.	93.3%	4.4%	2.2%	0%	97.8%	95.5%	4.5%	0%	0%	100%
53. Oral health status must be assessed periodically in all people with known or potential alterations of the oral cavity.	77.8%	17.8%	2.2%	2.2%	97.7%	97.7%	2.3%	0%	0%	100%
54. When compatible with the patient's clinical condition, the use of an electric toothbrush is preferable, as it is more effective at reducing plaque and biofilm compared with a manual toothbrush.	75.6%	11.1%	0%	13.3%	100%	77.3%	0%	0%	22.7%	100%
55. Accurate daily oral hygiene at home is suggested to reduce the risk of bacterial colonization and pulmonary complications.	97.8%	2.2%	0%	0%	100%	100%	0%	0%	0%	100%
56. Oral health and cleanliness of teeth, periodontal tissues, and oral mucosa must be maintained, as these factors facilitate bolus perception and processing during the oral preparatory phase.	93.3%	6.7%	0%	0%	100%	100%	0%	0%	0%	100%
57. The Free Water Protocol may be used in selected cases, always in conjunction with meticulous oral hygiene and exclusively in settings that ensure strict patient adherence.	64.4%	2.2%	2.2%	31.1%	96.8%	79.5%	2.3%	0%	18.2%	100%
**PATIENT AND CAREGIVER EDUCATION: DON'Ts**										

58. To ensure appropriate care delivery, it is suggested to avoid authority imbalances and friction related to “professional boundaries” that may arise from limited understanding of each other’s roles and responsibilities.	80%	6.7%	2.2%	11.1%	97.5%	88.6%	4.5%	2.3%	4.5%	97.6%
59. It is suggested not to use highly specialized scientific terminology without simple explanations, and not to overlook the health literacy level of the person with OD and their caregiver.	93.3%	4.4%	0%	2.2%	97.8%	97.7%	0%	2.3%	0%	97.7%
60. For adequate oral hygiene, the use of gels or mouthwashes alone is insufficient without the mechanical action of toothbrushing.	91.1%	2.2%	0%	6.7%	100%	100%	0%	0%	0%	100%
61. Do not use chlorhexidine-based mouthwashes for daily use, except in cases of gingivitis or periodontitis.	68.9%	4.4%	0%	26.7%	73.3%	77.3%	0%	0%	22.7%	100%

Note. The percentage of agreement for the sensitivity analysis reported in this Table was calculated as the proportion of ‘agree’ and ‘agree with reservations’ responses relative to respondents excluding those who selected the "unable to express an opinion" response. Therefore, this percentage does not match with the agreement reported in manuscript in Table 3, where the percentage of agreement was calculated as the proportion of ‘agree’ and ‘agree with reservations’ responses relative to all respondents, including those who selected the "unable to express an opinion" response.
