# Supplementary material for: Do’s and Don’ts in Mealtime Management for People with Oropharyngeal Dysphagia: A Modified Delphi Consensus Study from the Italian Society of Deglutology
Source: Nutrients. 2026 Oct 4;18(19):3268. doi: 10.3390/nu18193268 (PMC13666940; doi:10.3390/nu18193268)
Supplement: Supplementary file 1 [file nutrients-18-03268-s001.zip › Supplementary material S4. Item revision after Round 1 (clear).pdf]

Supplementary material

Table S1. Original and revised version of the statements modified after Round 1

Statement for Round 1	Statement for Round 2
4. It is suggested to conduct mealtime observations under the most ecologically valid conditions possible, namely in the person's usual environment, with foods and caregiver(s) that typically characterize their mealtime.	4. It is suggested to conduct mealtime observations under the most ecologically valid conditions possible, namely in the person's usual environment, **with the posture**, foods, and caregiver(s) that typically characterize their mealtime.
5. During mealtime observation, it is suggested to identify factors that act as barriers and facilitators.	5. During mealtime observation, it is suggested to identify **environmental and personal** factors that act as barriers and facilitators **for that specific person.**
7. Malnutrition screening should be performed in all patients with OD using validated tools such as the Mini Nutritional Assessment (MNA-SF) for the elderly and the Malnutrition Universal Screening Tool (MUST) or the Nutritional Risk Screening (NRS) 2002	7. Malnutrition screening should be performed in all patients with OD **using validated tools specific to the clinical setting, age group, and population**.
8. It is suggested to diagnose malnutrition through multidimensional assessment criteria such as the Global Leadership Initiative on Malnutrition (GLIM) criteria.	8. It is suggested to diagnose malnutrition through multidimensional assessment **criteria.**
9. It is suggested to use the tricepts skinfold thickness as a red flag for malnutrition in children with neurological disabilities	9. **To identify malnutrition in children with neurological disabilities, the presence of one or more of the following red flags should be investigated: 1. physical signs of undernutrition, such as pressure injuries, skin changes, or impaired peripheral circulation; 2. weight-for-age z-score < -2; 3. tricepts skinfold thickness <10th percentile for age and sex; 4. arm adipose/muscle area <10th percentile; 5. poor growth or failure to thrive.**
13. It is suggested not to perform an instrumental evaluation without a prior clinical assessment that clarifies the indications for the exam and suggests the most appropriate safety conditions.	13. It is suggested not to perform an instrumental evaluation **aimed at diet prescription** without a prior clinical assessment that clarifies the indications for the exam and suggests the most appropriate safety conditions.
14. During the mealtime observation, the clinician should not modify the mealtime conditions or provide instructions, except when necessary for the person's safety.	14. During the **first** mealtime observation, the clinician should not modify the mealtime conditions or provide instructions, except when necessary for the person's safety.
15. It is suggested not to rely on patient-, caregiver-, or parent-reported tools, as they cannot independently provide a comprehensive assessment of feeding skills, swallowing function, and mealtime behavior.	15. It is suggested not to rely **solely** on patient-, caregiver-, or parent-reported tools, as they cannot independently provide a comprehensive assessment of feeding skills, swallowing function, and mealtime behavior.
19. When controlling viscosity, it is important to consider the temperature, as a decrease in temperature may lead to thickening.	19. When controlling viscosity, it is important to consider the temperature **at which the food or beverage will be consumed**, as a decrease in temperature may lead to thickening.

21. To increase liquid viscosity, the use of xanthan-gum-based thickeners or other amylase-resistant formulations is suggested.	21. To increase liquid viscosity **in adults**, the use of xanthan-gum-based thickeners or other amylase-resistant formulations is suggested.
22. In case of OD for liquids and constipation, the use of fiber enriched PHGG gelled water is suggested.	22. In case of OD for liquids and constipation, the use of fiber-enriched PHGG gelled water **may be considered on an individual basis and in agreement with the treating physician.**
25. Before manipulating with the manipulation of solid dosage forms in a person with OD, it is suggested to evaluate whether an equivalent commercially available medication with marketing authorization can be prescribed in an alternative formulation or administered via a different route.	25. Before manipulating with the manipulation of solid dosage forms in a person with OD, **and always following a medical prescription**, it is suggested to evaluate whether an equivalent commercially available medication with marketing authorization can be prescribed in an alternative formulation or administered via a different route.
26.Do not neglect environmental (physical, social, cultural) factors related to mealtime.	26. Environmental (physical, social, cultural) factors **should be considered to identify intervention areas aimed at improving mealtime management.***
30. It is appropriate to verify and, if necessary, adjust posture before the meal so that they sit on the ischial tuberosities, and the feet are fully supported.	30**. If the person can safely maintain a seated position safely, with or without assistive devices**, it is appropriate to verify and, if necessary, adjust posture before the meal so that they sit on the ischial tuberosities, **the trunk is stable**, and the feet are fully supported.
31. In children with OD, an upright or slightly reclined posture is suggested to improve feeding safety and efficiency.	31. In children with OD, an upright or slightly reclined **trunk** posture is suggested to improve feeding safety and efficiency, **although this should be evaluated on a case-by-case basis.**
34. Do not feed a person with OD before checking and, if necessary, appropriately adjusting head and neck posture.	34. Do not feed a person with OD before checking whether head and neck posture **interferes with meal safety and efficiency** and, if necessary, appropriately adjusting it.
36. It is suggested to limit the use of distractions, such as television, during mealtimes.	36. It is suggested to limit the use of distractions, such as television, during mealtimes, **after verifying that they hinder safety and efficiency.**
37. Management of the familial and social implications of dysphagia should not be limited to the person with dysphagia alone, but must encompass the entire family and social context (family-centered care).	37. **In assessing and managing the implications of swallowing disorders, it is necessary to consider** the entire family and social context (family-centered care).*
40. The diet of older adults must have qualities that positively influence their quality of life.	40. The diet of older adults must have qualities that positively influence their quality of life. **Foods should be prepared with high-quality ingredients, in ways consistent with the diners' traditions, and presented in a hygienically safe and aesthetically pleasant setting.**
41. Foods for older adults should stimulate multisensory perception.	41. Foods **offered to** older adults should stimulate multisensory perception.
42. In people with artificial nutrition, it is suggested to verify whether small amounts of food can be consumed for hedonic purposes.	42. In people with **total** artificial nutrition, it is suggested to verify whether small amounts of food can be **safely** consumed for hedonic purposes, **while respecting personal preferences and tastes.**

44. Do not infer the nature of the family and social implications of OD solely from the severity and pathophysiology of the swallowing disorder.	44. Do not infer the nature **and extent** of the family and social implications of OD solely from the severity and pathophysiology of the swallowing disorder.
45. It is suggested to avoid authoritarian, indulgent, or uninvolved feeding styles.	45. It is suggested **that caregivers of children with OD** should avoid authoritarian, indulgent, or uninvolved feeding styles.
48. When educating the person with OD, it is suggested to verify learning by asking them to restate information in their own words and provide practical examples.	48. When educating the person with OD **and their informal caregivers**, it is suggested to verify learning by asking them to restate information in their own words and provide practical examples, **using communication modalities appropriate to the person's cognitive and linguistic abilities.**
49. When educating the person with OD, it is suggested to complement written informational materials with video resources, oral explanations, and in person demonstrations, while avoiding providing excessive information in in advance or concentrated in a single session.	49. When educating the person with OD **and their informal caregivers**, it is suggested to complement written informational materials with video resources, oral explanations, and in-person demonstrations, while avoiding providing excessive information in advance or concentrated in a single session.
50. In patients who present episodes of silent aspiration, it is suggested to assess the effectiveness of protective maneuvers (coughing, forced expiration) and investigate potential causes of ineffectiveness.	50. In patients who present episodes of silent aspiration, it is suggested to assess the effectiveness of protective maneuvers (coughing, forced expiration) **during meals** and investigate potential causes of ineffectiveness.
51. Food should not be administered in the absence of adequate oral hygiene.	51**. It is suggested to verify and ensure adequate oral hygiene before eating**. *
53. Oral health status must be assessed daily in all people with known or potential alterations of the oral cavity.	53. Oral health status must be assessed **periodically** in all people with known or potential alterations of the oral cavity.
54. The use of an electric toothbrush is preferable, as it is more effective at reducing plaque and biofilm compared with a manual toothbrush.	54. **When compatible with the patient's clinical condition**, the use of an electric toothbrush is preferable, as it is more effective at reducing plaque and biofilm compared with a manual toothbrush.
55. Accurate daily oral hygiene at home is suggested to reduce the risk of bacterial colonization.	55. Accurate daily oral hygiene at home is suggested to reduce the risk of bacterial colonization **and pulmonary complications.**
56. Oral health and cleanliness of teeth and periodontal tissues must be maintained, as these factors facilitate bolus perception and processing during the oral preparatory phase.	56. Oral health and cleanliness of teeth, periodontal tissues, **and oral mucosa** must be maintained, as these factors facilitate bolus perception and processing during the oral preparatory phase.
57. The Free Water Protocol may be used, always in conjunction with meticulous oral hygiene and exclusively in settings that ensure strict patient adherence.	57. The Free Water Protocol may be used **in selected cases**, always in conjunction with meticulous oral hygiene and exclusively in settings that ensure strict patient adherence.
58. It is suggested to avoid authority imbalances and friction related to "professional boundaries" that may arise from limited understanding of each other's roles and responsibilities.	58. **To ensure appropriate care delivery**, it is suggested to avoid authority imbalances and friction related to "professional boundaries" that may arise from limited understanding of each other's roles and responsibilities.

60. The use of gels or mouthwashes alone is insufficient without the mechanical action of toothbrushing.	60. **For adequate oral hygiene**, the use of gels or mouthwashes alone is insufficient without the mechanical action of toothbrushing.

* Statement moved from Don’ts to Do’s and reworded consequently

Note. Revisions to the statements are reported in bold. Other statements not cited in the table remained unchanged from Round 1 to Round 2
